# Long-read transcriptome landscapes of primary and metastatic liver cancers at transcript resolution

**DOI:** 10.1186/s40364-023-00554-w

**Published:** 2024-01-08

**Authors:** Zhiao Chen, Qili Shi, Yiming Zhao, Midie Xu, Yizhe Liu, Xinrong Li, Li Liu, Menghong Sun, Xiaohua Wu, Zhimin Shao, Ye Xu, Lu Wang, Xianghuo He

**Affiliations:** 1 Institutes of Biomedical Sciences, Shanghai Medical College, Fudan University Shanghai Cancer Center, Fudan University, 302 Rm., 7# Bldg., 270 Dong An Road, 200032 Shanghai, China; 2 Key Laboratory of Breast Cancer in Shanghai, Fudan University Shanghai Cancer Center, Fudan University, 200032 Shanghai, China; 3 Shanghai Key Laboratory of Radiation Oncology, Fudan University Shanghai Cancer Center, Fudan University, 200032 Shanghai, China; 4 Department of Hepatic Surgery, Fudan University Shanghai Cancer Center, Fudan University, 200032 Shanghai, China; 5 Department of Pathology, biobank, Fudan University Shanghai Cancer Center, Shanghai, China; 6 Department of Gynecologic Oncology, Fudan University Shanghai Cancer Center, Fudan University, 200032 Shanghai, China; 7 Department of Breast Surgery, Fudan University Shanghai Cancer Center, Fudan University, 200032 Shanghai, China; 8 Department of Colorectal Surgery, Fudan University Shanghai Cancer Center, 200032 Shanghai, China

**Keywords:** Third-generation sequencing technology, Specific RNA transcript, Isoform switch, Metastasis-specific transcript, Immunosuppressive environment

## Abstract

**Background:**

The liver ranks as the sixth most prevalent site of primary cancer in humans, and it frequently experiences metastases from cancers originating in other organs. To facilitate the development of effective treatments and improve survival rates, it is crucial to comprehend the intricate and diverse transcriptome landscape of primary and metastatic liver cancers.

**Methods:**

We conducted long-read isoform sequencing and short-read RNA sequencing using a cohort of 95 patients with primary and secondary liver cancer who underwent hepatic resection. We compared the transcriptome landscapes of primary and metastatic liver cancers and systematically investigated hepatocellular carcinoma (HCC), paired primary tumours and liver metastases, and matched nontumour liver tissues.

**Results:**

We elucidated the full-length isoform-level transcriptome of primary and metastatic liver cancers in humans. Our analysis revealed isoform-level diversity in HCC and identified transcriptome variations associated with liver metastatis. Specific RNA transcripts and isoform switching events with clinical implications were profound in liver cancer. Moreover, we defined metastasis-specific transcripts that may serve as predictors of risk of metastasis. Additionally, we observed abnormalities in adjacent paracancerous liver tissues and characterized the immunological and metabolic alterations occurring in the liver.

**Conclusions:**

Our findings underscore the power of full-length transcriptome profiling in providing novel biological insights into the molecular mechanisms underlying tumourigenesis. These insights will further contribute to improving treatment strategies for primary and metastatic liver cancers.

**Supplementary Information:**

The online version contains supplementary material available at 10.1186/s40364-023-00554-w.

## Background

Liver cancer is the fourth leading cause of cancer-related mortality worldwide. Hepatocellular carcinoma (HCC) comprises 80–90% of primary liver cancers and typically develops in the context of cirrhosis and inflammation [1]. Liver metastasis commonly occurs in various malignancies, including colorectal cancer (CRC), pancreatic cancer, melanoma, lung cancer and breast cancer, with CRC being the most frequent primary cancer that metastasizes to the liver [2]. The presence of liver metastasis is associated with a significant decrease in the 5-year survival rate and diminished quality of life [3]. While extensive cancer sequencing efforts have focused on primary tumours such as HCC, revealing diverse molecular regulators of this intricate multistep process [4, 5], our understanding of matched primary tumour and hepatic metastasis transcriptomic profiles in patients remains limited. Furthermore, the short-read lengths (100–200 bases) of conventional short-read RNA sequencing hinder direct inference of full-length transcript structures [6]. To facilitate the development of effective treatments and improve survival outcomes, it is crucial to comprehend the intricate and diverse molecular mechanisms underlying primary and metastatic liver cancers.

Long-read sequencing enables comprehensive transcriptome analysis by identifying full-length splice isoforms and various posttranscriptional events [7, 8]. By utilizing long-read SMRT sequencing, a study investigated eight patient-derived HCC cases and the Hep3B cell line, revealing the prevalence of alternative isoforms and tumour-specific isoforms resulting from aberrant splicing during liver tumourigenesis [9]. Another investigation employed Nanopore RNA-seq for tumours, matched portal vein tumour thrombi, and peritumoural tissues from three HCC patients, leading to the discovery of two novel prognostic transcripts [10]. Recently, one study developed an analysis pipeline utilizing the Oxford Nanopore sequencer, unveiling novel splicing abnormalities and oncogenic transcripts in liver cancer [11]. While these studies have shed light on the importance of alternative isoforms in HCC development, a comprehensive depiction of the entire alternative isoform landscape in HCC remains challenging, and exploration of the full-length transcriptomes of primary and metastatic liver cancers is limited.

Accurate quantification of transcripts using long-read sequencing requires the depth of coverage, which is prohibitively expensive. Therefore, in this study, we adopted a cost-effective approach by combining long-read sequencing, specifically isoform sequencing (Iso-Seq), with short-read RNA sequencing. This hybrid approach allowed us to survey the transcriptome landscapes of primary and metastatic liver cancers. Specifically, we systematically investigated HCC, paired primary tumours (PTs) and liver metastases (LMs), as well as matched nontumour liver tissues (LM-NTs). Our study presents the first full-length transcriptome profiles of primary and metastatic liver cancer at transcript resolution, and the findings will be useful for understanding the molecular basis of liver cancer and will further transform the approach to treating primary and secondary liver cancers.

## Methods

### Patients and sample characteristics

We obtained samples of liver metastases that had been pathologically diagnosed from Fudan University Shanghai Cancer Center. Written informed consent was obtained from the patients, and the study was approved by the Ethics Committee of the Fudan University Shanghai Cancer Center (approval number: 2011-ZZK-33). The study was conducted in accordance with ethical guidelines outlined in the Declaration of Helsinki. In total, we collected 177 samples from 95 patients, including 23 samples from patients with HCC, 7 samples from patients with nasopharyngeal carcinoma, 12 samples from patients with breast cancer, 5 samples from patients with gastric cancer, 3 samples from patients with kidney cancer, 5 samples from patients with neuroendocrine tumour, 20 samples from patients with colorectal cancer, 18 samples from patients with ovarian cancer, and 2 samples from patients with cervical cancer. These samples underwent Iso-seq. Additionally, we performed RNA-seq on 203 samples from the same patients. Immunohistochemistry analysis was conducted on 62 liver tissues from 62 patients, including 2 samples from patients with nasopharyngeal carcinoma, 6 samples from patients with breast cancer, 1 sample from a patient with kidney cancer, 20 samples from patients with colorectal cancer, 11 samples from patients with ovarian cancer, 3 samples from patients with cervical cancer, and 19 samples from patients with hepatic hemangioma.

### PacBio library and single-molecule sequencing

RNA samples of clinical tissue specimens were extracted using TRIzol reagent (Invitrogen, CA, USA). The purity and contamination of the RNA were assessed through UV-spectrophotometry using a Nanodrop spectrophotometer. The quality of the input total RNA samples was evaluated by measuring the RNA Integrity Number (RIN) and concentration using an Agilent 2100 instrument. Only RNA samples with a RIN above 7.0 were used for library construction. Full-length cDNA synthesis from transcripts containing poly-A tails was generated from 2 µg of total RNA per sample using the Clontech SMARTer PCR cDNA Synthesis Kit (catalog# 634,925 or 634,926) according to PacBio’s Iso-Seq Template Preparation for the Sequel System. SMRTbell libraries were constructed using the SMRTbell™ Template Prep Kit (Pacific Biosciences, Part No. 100-259-100), then sequenced on the PacBio Sequel II System (BerryGenomics, Beijing, China).

### PacBio data analysis

The sequence data were processed using the SMRT Analysis software (IsoSeq 3) in PacBio SMRT Analysis v6.0 to obtain high-quality, full-length transcript sequences, followed by downstream analysis. Sequence was processed by using Iso-Seq (version 3.4.0) workflow to generate full-length reads [12]. Briefly, raw data of sub-reads were merged to circular consensus sequence (CCS) reads with minimum predicted accuracy in 0.9. Full length reads were generated through removal of 5’ and 3’ cDNA primers by using lima with default parameters. Artificial concatemers reads and polyA tails were then removed by using isoseq3 refine to generate full-length, non-chimeric (FLNC) reads. FLNC reads were then clustered into high-quality transcripts by using isoseq3 cluster. Filtered transcripts were mapped to the human genome (hg38, GENCODE v.44) using minimap2 (version 2.17) [13]. Isoforms were subsequently collapsed and correlated using cDNA_Cupcake (version 19.0.0) and SQANTI3 (version 5.1.1) [14]. In the analysis of SQANTI3 output and based on user-defined rules, all transcript junctions must be identified in RNA-seq data. Additional filters are applied: full-splice match (FSM, no filter - all included), incomplete-splice match (ISM, filtered out based on 3’ or 5’ end incompleteness, respectively). For ISM, novel in catalogue (NIC), novel not in catalog (NNC), and others transcripts, filtering is applied when there are 16 or more adenines within the 20 bp downstream of the annotated Transcription Terminating Site (TTS) at the genomic level. Transcripts from all samples were merged into a single non-redundant GTF file using gffcompare (version 0.11.2) [15] and annotated using gffcompare and SQANTI3. Next, we filtered the transcripts without junction support from RNA-Seq. Finally, transcripts detected in at least two biological replicates by Iso-Seq were used for GTF reference for RNA-seq quantification.

### RNA-seq

For RNA-seq, the same RNA samples from clinical tissue specimens (used for single-molecule sequencing) and Huh 7 cells were extracted using TRIzol reagent (Invitrogen, CA, USA). The quality of the input total RNA samples was assessed by measuring the RNA Integrity Number (RIN) and concentration using an Agilent 2100 instrument. Only RNA samples with a RIN above 7.0 were used for library construction. The sequencing library was prepared using the VAHTSTM Total RNA-seq Library Prep Kit for Illumina (Vazyme, Nanjing, China) and subjected to paired-end sequencing on the Illumina HiSeq platform. The paired-end reads were aligned to hg38 reference genome using STAR [16]. The resulting BAM files from STAR were converted into bedGraph format using BEDTools and further transformed into BigWig files for visualization with UCSC Genome Browser or IGV [17]. Raw gene expression levels (raw read counts) were calculated using FeatureCounts [18], and gene expression was measured in transcripts per million (TPM). RNA-seq was performed on 23 pairs of HCCs and adjacent nontumorous livers using a polyA-selected strategy. Data for 371 HCC tissues and 50 adjacent non-cancerous samples were obtained from The Cancer Genome Atlas (TCGA; https://portal.gdc.cancer.gov) with official authorization. Raw BAM files downloaded from the Genomic Data Commons (GDC) data portal were converted into FASTQ files using bedtools (version 2.29.2) bamtofastq. Additionally, raw RNA-seq data (FASTQ files) of 188 HCCs and matched nontumorous livers were downloaded from Gene Expression Omnibus (GEO) under accession numbers GSE77314 [19], GSE94660 [20], GSE124535 [21] and GSE138485 [22]. Public RNA-seq data for colon and breast cancer metastasis to the liver were retrieved from the GEO database and the database of Genotypes and Phenotypes (dbGaP) under the following accession numbers: GSE50760 [23], GSE92914 [24], GSE179979, GSE58708 [25] from GEO, and phs000673 [26] from dbGaP. Transcript expression levels, measured in TPM, were quantified by mapping RNA-seq reads to the GTF reference generated from long-read isoforms using Salmon (version 1.5.2) [27] in mapping-based mode. After normalizing the RNA-seq data using a log2 (TPM + 1) transformation, the Combat method from the R package “surrogate variable analysis (SVA)” was applied to correct for batch effects between the datasets from different studies [28].

### t-distributed stochastic neighbor embedding (t-SNE) analysis

To prepare for t-SNE analysis using the Rtsne package in R, we applied preprocessing steps to the isoform expression data. These steps involved log-transforming the expression values to stabilize variances and scaling them to have a mean of 0 and a standard deviation of 1. The purpose of these preprocessing steps was to enhance the quality and comparability of the data. Following the preprocessing, the t-SNE analysis was conducted to visualize the relationships between samples in a reduced-dimensional space.

### Differential expression analysis and pathway enrichment analysis

To assess the statistical differences between tumour and paired normal samples, we employed the Wilcoxon rank-sum test. Genes or transcripts were considered significantly differentially expressed if their absolute log2 fold change (|log2(FC)|) was greater than 1 and the false discovery rate (FDR) was less than 0.05. In order to avoid any potential confounding effects on subsequent analyses, we carefully removed incomplete-splice match (ISM) transcripts, which may arise due to RNA degradation or incomplete reverse transcription. Functional enrichment and gene set variation analyses were performed using the R package “clusterProfiler” (version 3.16.1) [29], as previously described [30]. For pathway enrichment analysis using lists of differentially expressed transcripts (DETs), we collapsed the transcripts to genes before conducting the analysis.

### Specific RNA transcripts and isoform switching analysis

HCC-SRTs (Hepatocellular Carcinoma-Specific RNA Transcripts) are defined as transcript variants found exclusively in HCC samples and not in HCC-NT (nontumor) samples. The identification of HCC-SRTs through RNA-seq follows the following criteria:

(i) The median expression level of the transcript in HCC is at least 10-fold higher than the maximum expression level observed in all adjacent normal tissues within the datasets.
(ii) The transcript is expressed (TPM > 0.5) in more than 5% of tumour samples.

Regarding isoform switching events, they are defined as cases where, compared to the adjacent normal tissues, at least one transcript variant of the same gene is upregulated while another transcript variant is downregulated in HCC samples.

### Isoform-based clustering

For isoform-based clustering, we employed k-means clustering by the R package “ConsensusClusterPlus” (version 1.52.0). Empirical cumulative distribution CDF plots were generated to determine the optimal number of isoform-based HCC subtypes.

### Motif enrichment analysis

To identify potential transcription factors that regulate the transcription of these transcripts, we conducted motif enrichment analysis using MEME with default parameters. Motifs were identified in the upstream region (-1000 nucleotides) and downstream region (+ 1000 nucleotides) of the transcription start sites.

### Gene set enrichment analysis (GSEA)

Following the knockdown of SP1, all transcripts were quantified and ranked based on their fold change. We performed GSEA using the R package “clusterProfiler” to analyze the ranked transcripts. To identify the expression of SRTs that are correlated with SP1 expression in HCC tumour samples, GSEA was performed on the ranked SRTs based on the Spearmen’s correlation coefficient with SP1. Additionally, to identify isoform switch profiles that correlate with tissue-specific transcript patterns in HCC and matched nontumor liver samples, we ranked the transcripts based on the log2-fold change of the TPM values between HCC and HCC-NT samples. Subsequently, GSEA were performed on liver-specific and testis-specific transcripts, as defined by the GTEx database.

### Machine learning based on random forest

The harmonized colorectal cancer liver metastases (CRLM, from our Iso-seq and GEO datasets) and CRC patients (COAD and READ from TCGA) (n = 713) were randomly divided into training (n = 570) and held-out testing (n = 143) sets at a ratio of 4:1. Both sets had the same distribution of cancer types. We selected 26 SRTs and employed the random forest model using the R package “randomForest” (version 4.6.14) with 5-fold cross-validation on the training set. We then used the same parameters to predict the testing cohort. The performance of the machine learning model based on SRTs was assessed using receiver operating characteristic (ROC) curve. For breast cancer liver metastases (BCLM, from our Iso-seq and GEO datasets) and breast cancer (BRCA from TCGA) cohorts, harmonized patients (n = 1093) were randomly divided into training (n = 874) and held-out testing (n = 219) sets. Ten SRTs were used to construct the random forest model. The same procedure was followed to determine tumour tissue of origin (TOO) models.

### Statistical analysis

We used the Wilcoxon rank-sum test to determine statistical differences between two groups. Multiple testing corrections were performed using the Benjamini and Hochberg method. Kaplan–Meier survival curves were generated using the R package “survminer” (version 0.4.8), and Log-rank tests were utilized to compare the overall survival differences between groups with the R package “survival” (version 3.2–3). The chi-squared (χ2) test was employed to assess differences in transcript distributions across five categories (FSM, ISM, NIC, NNC, and others) between indicated groups as well as the expression of PD-1 and PD-L1 between liver tissues from LM patients and hemangioma patients. Statistical analyses were performed using SPSS (IBM, NY, USA) and the R project. A p-value < 0.05 was considered statistically significant.

## Results

### Long-read transcriptome landscapes of primary and metastatic liver cancers

**Fig. 1:**
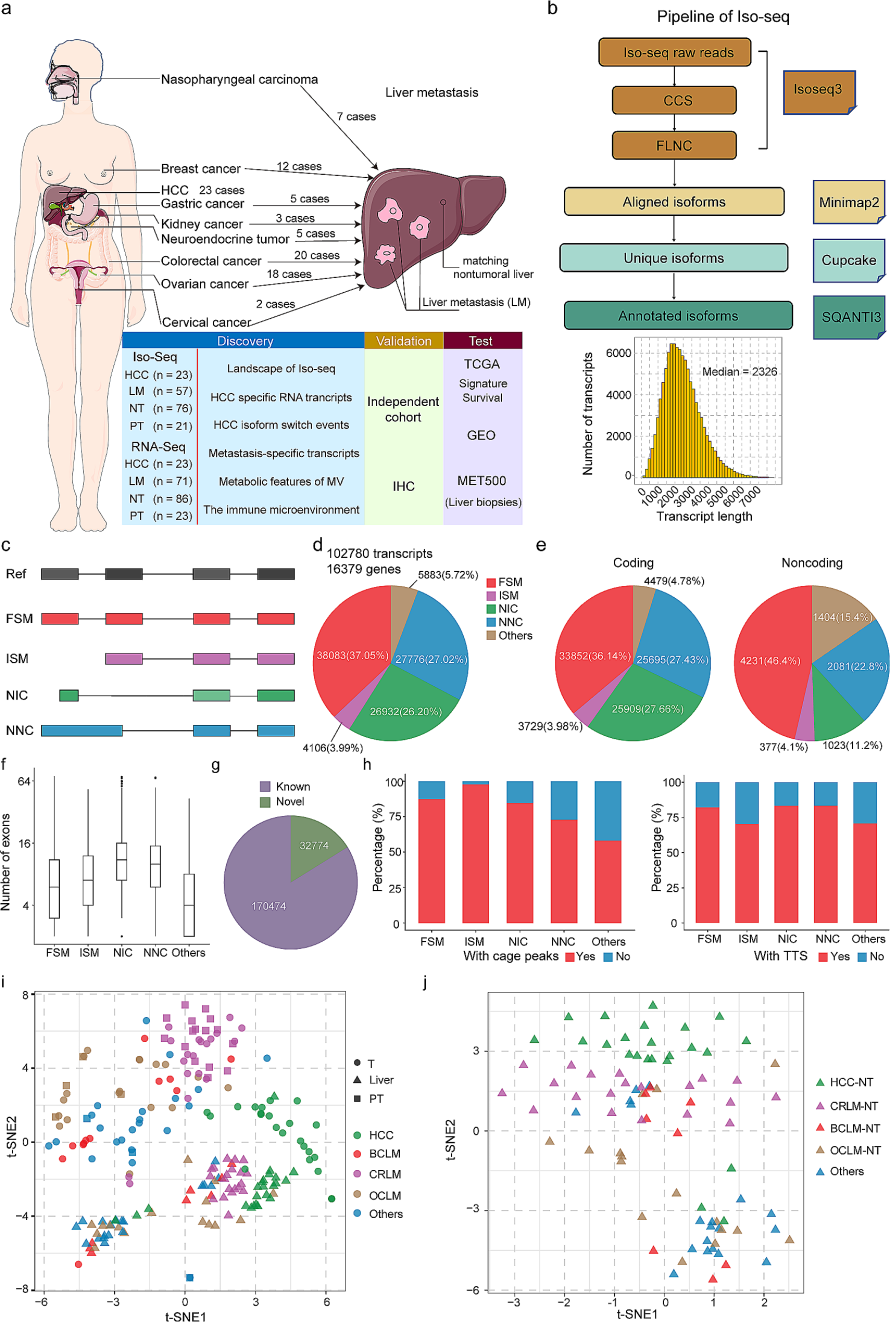
Landscape of long-read transcriptomes in primary and metastatic liver cancers. **a**. Schematic representation of primary and metastatic liver cancer isoform profiling using Iso-seq and RNA-seq. **b**. Isoform calling algorithm employed for Iso-Seq data analysis. **c**. Types of isoforms identified and their graphical illustration. (**d**-**e**). The percentage and number of distinct isoforms in each category from (**c**) are indicated, including total, coding, and noncoding transcripts. **f**. Characteristics of novel (NIC and NNC) and known (FSM and ISM) transcripts. NIC and NNC isoforms have more exons. **g**. Proportion of annotated and unannotated junctions based on comparison with reference transcripts. **h**. Percentage of Iso-seq isoform transcription start sites supported by CAGE (FANTOM5) or transcription termination sites supported by the presence of a poly(**A**) motif (SQANTI3), plotted per category from (**c**). **i**. Global transcript expression patterns of primary and metastatic liver cancers visualized using a t-distributed stochastic neighbor embedding (t-SNE) projection. The position of samples within the plot reflects the relative similarity in transcript expression. Samples are color-coded on the basis of their assigned analysis cohort. T, tumour samples, including primary and metastatic liver cancer samples; Liver, nontumor liver tissues; PT, primary tumour samples of metastatic liver cancer. **j**. Global transcript expression patterns of liver samples depicted by a t-SNE projection, including HCC-NT, and LM-NT samples

This study included a cohort of 95 patients with primary and secondary liver cancer who underwent hepatic resection. Among the patients, there were 41 males (43.2%) and 54 females (56.8%). The median age of the cohort was 55 years, ranging from 31 to 77 years (Table S1). Figure 1a illustrates the cancer types represented in the cohort. The top three liver metastasis cancer types observed in our cohort were colorectal cancer liver metastases (CRLM), breast cancer liver metastases (BCLM), and ovarian cancer liver metastases (OCLM). A total of 203 tissues were collected from these patients, including paired HCC and adjacent nontumor tissues (HCC-NT), paired primary tumours and liver metastases, and matched nontumor liver tissues. Short-read sequencing successfully evaluated 203 tissues; Iso-Seq analysis was performed on 177 tissues. For more detailed information, please refer to Table S1.

The isoforms obtained through single-molecule real-time circular consensus sequencing were polished using the ToFU (Transcript Isoforms Full-length and Unassembled) pipeline (Fig. 1b). Overall, we identified 102,780 unique FL transcript isoforms spanning 16,379 annotated genes in liver cancer, with an average isoform length of 2.3 kb (Fig. 1b). These isoforms were classified into five categories based on their junction match to a reference transcriptome (GENCODE v.44) using SQANTI3 (Fig. 1c).

Among the isoforms, 37.05% (38,083) were classified as full-splice matches (FSMs), indicating a perfect match to known transcripts. Additionally, 3.99% (4,106) were categorized as incomplete-splice matches (ISMs), representing transcripts that are present in the reference but lack either the 5’ region, 3’ region, or both. Furthermore, 26.2% (26,932) and 27.02% (27,776) were classified as novel in catalogue (NIC; a combination of known splice donors or acceptors that have not been previously catalogued in the same transcript) and novel not in catalogue (NNC; at least one splice site not present in reference), respectively. The remaining isoforms included antisense and intergenic transcripts (Fig. 1d).

The distributions of transcripts varied between primary cancer and liver metastasis tissues (chi-squared test, *p* < 0.001) and between HCC and matched nontumor liver tissues (chi-squared test, *p* < 0.001). Additionally, the distributions of isoforms differed based on the origin of primary cancers (chi-squared test, *p* < 0.001) (Fig. S1a-c). Most of the transcripts are protein-coding, and their distribution differed from that of noncoding transcripts (chi-squared test, *p* < 0.001). Specifically, of the protein-coding and noncoding transcripts, 36.14% and 46.4% were found to be FSM, 3.98% and 4.1% ISM, 27.66% and 11.2% NIC, and 27.43% and 22.8% NNC, respectively (Fig. 1e). Compared to known isoforms, novel transcript isoforms (both NIC and NNC) exhibited a higher number of exons (Fig. 1f and S1d). Notably, 32,774 junctions are not annotated in GENCODE v.44 (Fig. 1g).

To assess the reliability of the full-length isoforms, we utilized various quality features provided by SQANTI3, including functional genomic evidence such as overlap of 5′ transcript ends with independently published cap analysis of gene expression (CAGE) data and 3′ ends with polyA tails (Fig. 1h). Moreover, the number of detected isoforms in samples correlated with the presence of full-length read nonchimeric (FLNC) sequences (Fig. S1e). We also compared the distribution of transcripts based on the protein-coding and noncoding transcripts within each group. Overall, novel isoforms accounted for 19.3–28.7% of the sequenced transcripts in each group (average = 23.3%), and noncoding transcripts were shorter in length than coding RNA transcripts (Fig. S1f-o).

To evaluate the extent of tissue- and cancer-specific gene expression maintenance across primary and metastatic lesions, we utilized t-distributed stochastic neighbourhood embedding (t-SNE) projection to visually analyse isoform expression patterns. It was evident that tumours exhibited significantly different dissimilarity compared to nontumor liver tissues, including nontumor liver tissues from both HCC and metastatic liver cancer patients. Furthermore, HCC samples were distinctly separated from liver metastasis samples. However, compared with HCC, metastatic samples showed less pronounced separation based on the type of primary cancer. In addition, most primary and matched metastatic samples did not segregate based on the primary cancer type (Fig. 1i). Most importantly, the nontumor liver tissues from liver metastasis and HCC patients demonstrated clear segregation from each other, which indicated that the environment of the healthy liver may be modified for transformation of HCC or metastatic cells from extrahepatic malignancies (Fig. 1j). Collectively, these findings indicate that isoform expression displays tissue-specific patterns and transcript diversity.

### Profiling of significantly differentially expressed RNA transcripts in primary Liver cancer

To examine the biological and clinical significance of isoforms in HCC, we initially quantified expression of isoforms in HCC and HCC-NT. To account for batch effects between our Iso-seq HCC samples and previously published nonmetastatic HCC cases, we utilized the Combat method from the R package surrogate variable analysis (SVA) for batch effect correction (see Methods). To evaluate the consistency between our current study and previously published nonmetastatic HCC cases, we performed clustering and t-SNE analysis on HCC and HCC-NT samples from various datasets to assess similarity in transcript expression profiles. Upon clustering individual samples, we observed that they tended to cluster based on their cancerous or nontumor nature, rather than being grouped based on the different dataset sources (Fig. S2a). Furthermore, t-SNE analysis revealed that although the samples derived from different datasets, cancerous tissue samples clustered together, as did nontumor tissue samples, without exhibiting distinct differences between the different dataset sources. Notably, clear differences were observed between cancerous and nontumor tissue samples (Fig. S2b). These findings indicate that the HCC and HCC-NT data from our study and previously published nonmetastatic HCC cases show similarities in their transcript expression patterns.

**Fig. 2:**
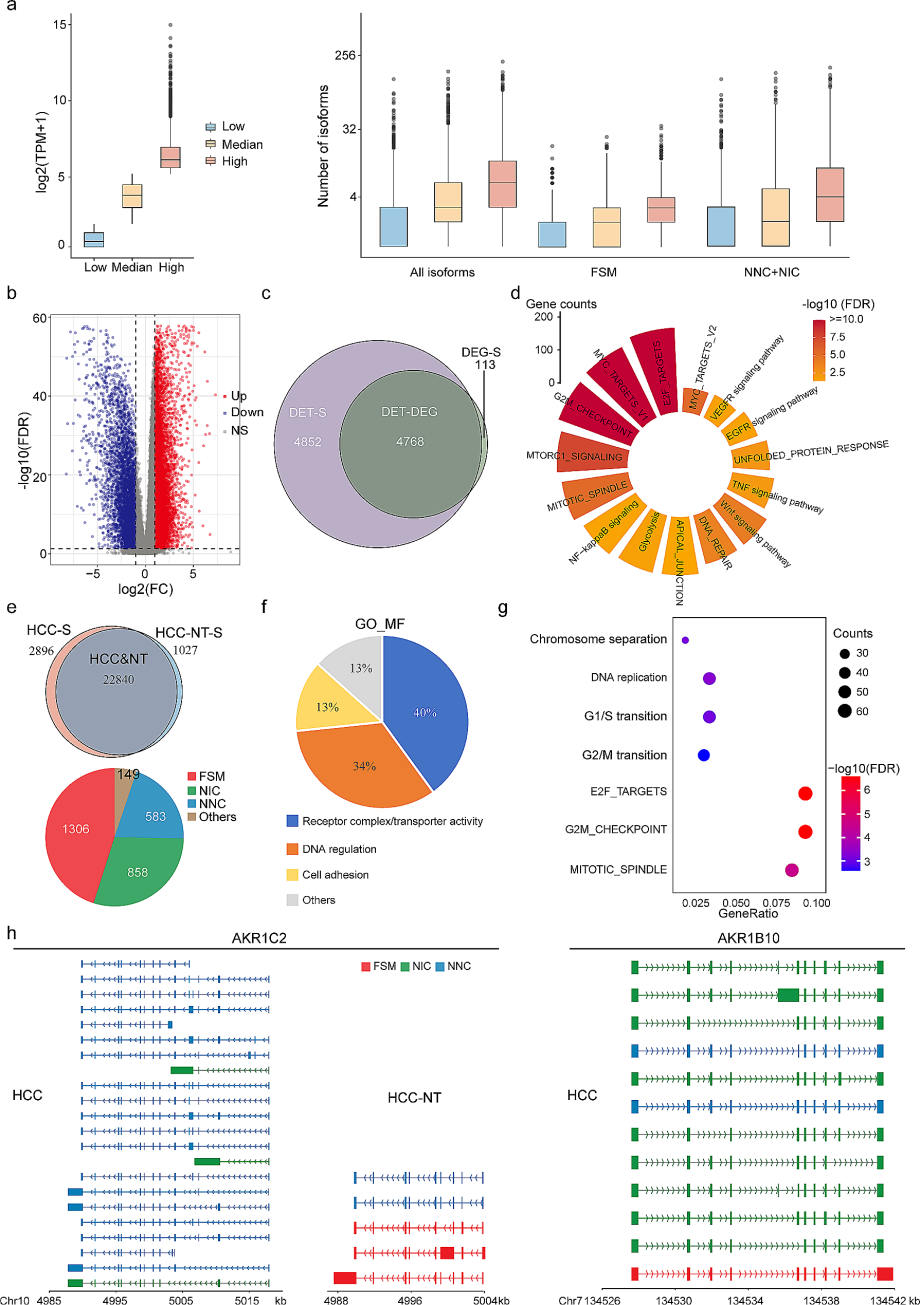
Profiling of significant differentially expressed RNA transcripts between primary HCC and matched nontumor liver tissues. **a**. Correlation between gene expression levels obtained from RNA-seq and the number of transcript isoforms detected by Iso-seq. Genes are grouped into quartiles based on expression levels: low (first quartile), median (second and third quartiles), and high (fourth quartile). **b**. Volcano plot illustrating DETs in HCC and HCC-NT. Red and blue dots represent significantly upregulated and downregulated transcripts (FDR < 0.05), respectively. **c**. Venn diagram showing the overlap between DETs and DEGs between HCC and HCC-NT. DET-s, DET-specific. DEG-S, DEG-specific. **d**. Pathway enrichment analysis for genes associated with DETs. **e**. Top, Venn diagram indicating the number of common and SRTs in HCC and HCC-NT samples. SRTs are defined as a fold change larger than 10 between tumour and paired adjacent nontumor tissues. HCC-S, HCC-specific. HCC-NT-S, HCC-NT specific. Bottom, distribution of isoform numbers for HCC SRTs. **f**. GO analysis of HCC SRT-associated genes. **g**. Pathway enrichment analysis of HCC SRTs. **h**. Structure of AKR1C2 and AKR1B10 transcripts in HCC and HCC-NT samples

We categorized genes into three groups based on their RNA-seq expression levels, low, average, and high, using TPM cut-offs. We observed that the average number of detected total isoforms and novel isoforms (NIC + NNC) increased for genes expressed at low, average, and high levels. Interestingly, there were detectable isoforms even for genes with low expression levels, including novel isoforms (Fig. 2a). These data indicate that Iso-Seq technology is capable of capturing transcripts even for genes with low expression, and that the NIC and NNC isoforms identified in our Iso-Seq data are expressed at appreciable levels.

When comparing gene expression profiles between tumour and adjacent nontumor tissues, we identified 26,763 differentially expressed transcripts (DETs) after adjusting for multiple testing (Fig. 2b). To further elucidate the significance of gene versus isoform expression patterns, we examined differentially expressed genes (DEGs) with no significant isoform expression changes and DETs with no significant gene expression changes (Fig. S2c). The Venn diagram in Fig. 2c illustrates the overlap between DETs and DEGs at the gene level. Interestingly, only 2.3% (113 of 4,881) of DEGs showed no change in isoform distribution, whereas over half (50.4%) of DETs were among genes with no significant gene expression changes (Fig. 2c and Table S2). These DETs, which may have been previously overlooked due to comparable gene expression levels, represent an additional dimension in the analysis of differential expression.

To gain insights into the functional implications of DETs, we performed pathway enrichment analysis using the Kyoto Encyclopedia of Genes and Genomes (KEGG) and MSigDB Hallmark databases [31] for genes associated with DETs. We found that spliced genes with novel isoforms were strongly associated with key pathways in HCC, which were overrepresented, including replication (Myc targets and G2M checkpoint pathway), NF-κB signalling, mTORC1 signalling, and Wnt signalling pathways et al. (Fig. 2d). These findings highlight the potential functional relevance of the novel isoforms and their involvement in critical pathways associated with HCC.

Subsequently, we aimed to investigate the specific RNA transcripts (SRTs) expressed in HCC but not in HCC-NT. We identified a total of 2,896 SRTs in HCC, comprising 2,695 protein-coding RNAs and 201 noncoding RNAs, with approximately half of them being novel transcripts (Fig. 2e and Table S3). Gene Ontology (GO) analysis of these HCC SRTs revealed that 87% of the enriched molecular function terms are associated with receptor complex/transporter activity, DNA regulation, and cell adhesion (Fig. 2f). Pathway enrichment analysis demonstrated these isoforms to be enriched in pathways known to be deregulated in HCC, such as the cell cycle, E2F targets, and mitotic spindle (Fig. 2g). Individual oncogenes (oncogene lists obtained from MSigDB [31]) with a high gain of novel isoforms in the Iso-seq cancer transcriptome were identified; for example, AKR1C2 and AKR1B10 are often overexpressed in HCC and contribute to hepatocarcinogenesis. In addition to the isoforms annotated in GENCODE v.44, we observed a 2.2-fold increase for AKR1C2 and a 2.75-fold increase for AKR1B10 in NIC + NNC isoforms (Fig. 2h).

### Clinical significance and regulation of SRT expression in primary Liver cancer

**Fig. 3:**
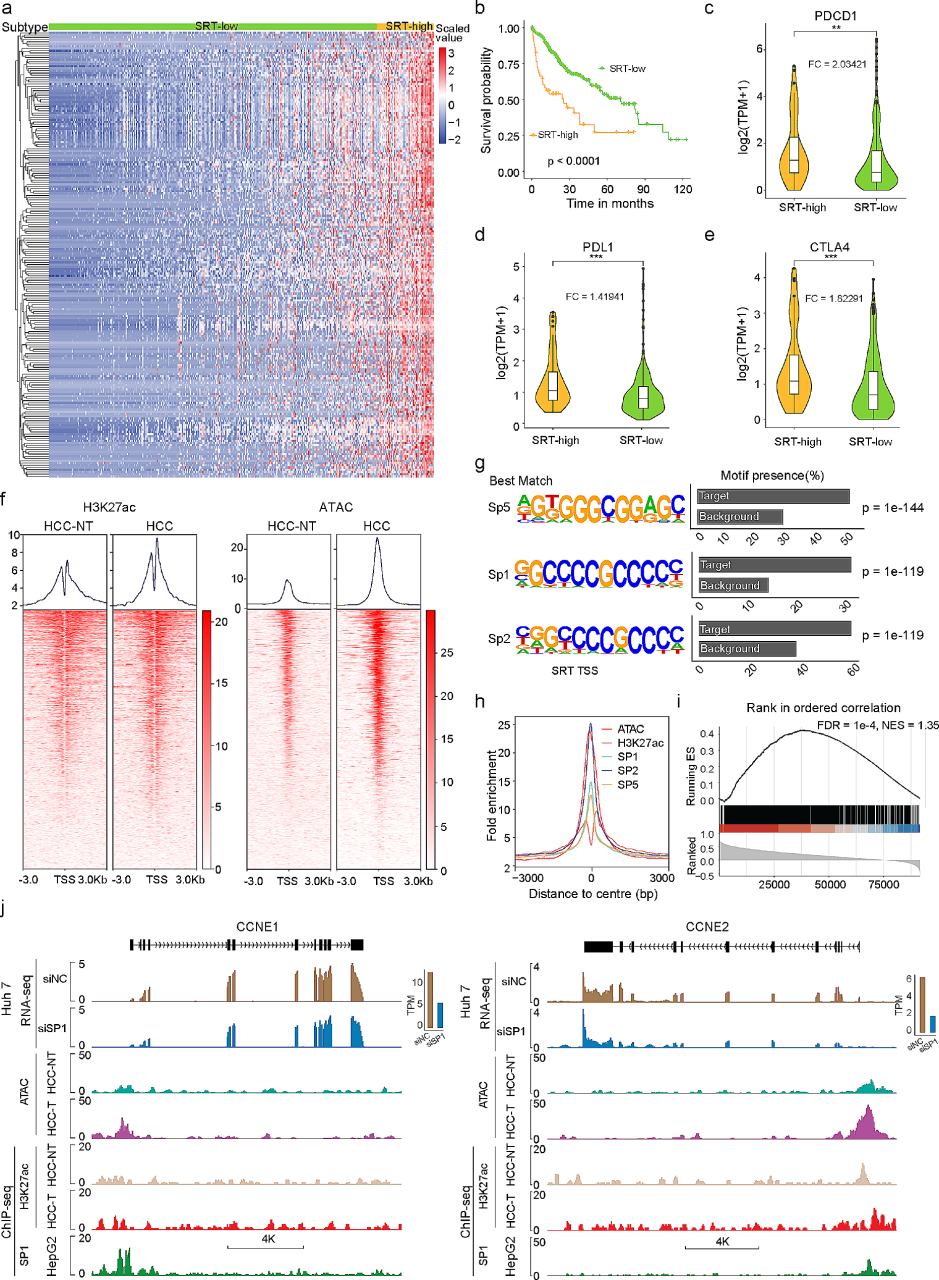
Clinical significance and expression control of the SRTs in primary liver cancer. **a**. Unsupervised clustering of patient samples based on the expression profiles of the HCC SRTs. **b**. Kaplan-Meier curves illustrating overall survival in SRT-high (red) and SRT-low (green) patients in the TCGA-LIHC cohort. **c**-**e**. Differences in the expression of immune checkpoint inhibitors between the SRT-high and SRT-low groups. Statistical significance between the two groups was determined using the Wilcoxon rank-sum test. FC, fold change (High Versus Low). **f**. Enrichment of ChIP-seq peaks for H3K27ac and ATAC-seq peaks within 3 kb from the TSSs of HCC SRTs in HCC and HCC_NT. **g**. Top enriched DNA binding motifs with significant P values identified through de novo analysis of sequences within 0.5 kb from the TSSs of HCC SRTs. **h**. Line plots displaying ATAC-seq and ChIP-seq signals of H3K27ac, SP1, SP2, and SP5 centered at the most enriched motif of SRTs. **i**. GSEA of HCC SRTs. Transcripts are ranked based on the correlation between the expression of SP1 and SRTs. The Normalized Enrichment Score (NES) and FDR are provided. **j**. Profiles of SP1 and H3K27ac occupancy, as well as ATAC-seq peaks, at the promoter regions of CCNE1 and CCNE2 in liver cancer cell, HCC, and HCC-NT tissues. **p < 0.01, ***p < 0.001

To assess the clinical relevance of the HCC-SRTs, we employed consensus clustering to classify patients into two subtypes based on SRT expression profiles. After unsupervised clustering, 55 of 371 HCC patients (14.8%) from the TCGA dataset were identified as the SRT-high subtype, and the remaining 316 patients as the SRT-low subtype (Fig. 3a). As expected, patients in the SRT-high subtype exhibited significantly worse prognosis and overall survival (Fig. 3b). Subsequently, we aimed to investigate the functional impact of SRT events associated with cell proliferation, the epithelial-mesenchymal transition (EMT), immune checkpoint inhibitor expression, and cancer hallmarks such as cancer cell stemness, angiogenesis, anti-apoptosis, glycolysis, hypoxia, and inflammation. The results demonstrated significant upregulation of PDCD1 (PD-1), CD274 (PD-L1), and CTLA4 in the SRT-high subtype compared to the SRT-low subtype (Fig. 3c-e). SRT events showed positive correlations with most cancer hallmarks, including cell proliferation, stemness, glycolysis, hypoxia, and inflammation (Fig. S3a-b). However, no associations were found between SRT events and EMT or angiogenesis. These findings suggest that SRTs are associated with poor prognosis, tumour microenvironment infiltration of the immune response, and enrichment of cancer hallmarks.

To investigate the mechanisms underlying the generation of these SRTs, we analysed the genomic locations of SRTs in comparison to annotated genes. Interestingly, we observed that the vast majority of SRTs overlap with known genes, with over 70% of them containing multiple TSSs (Fig. S3c). Epigenetic events, such as chromatin accessibility and histone modifications, have been shown to be closely associated with expression levels of tumour-specific transcripts [32, 33]. Thus, we employed chromatin immunoprecipitation sequencing (ChIP-seq) and an assay for transposase-accessible chromatin (ATAC) sequencing to investigate variations in promoter landscapes between HCC and HCC-NT. Analysis using an H3K27ac antibody revealed enriched H3K27ac deposition and increased chromatin accessibility in genome regions encoding SRTs in HCC compared to HCC-NT (Fig. 3f). To determine involvement of key transcription factors in SRT generation, we conducted de novo binding motif analysis on gained promoters in HCC samples. Strikingly, motifs for the specificity protein (Sp) family, including SP1, SP2, and SP5, were highly enriched in gained promoters in HCC (Fig. 3g). The Sp family of proteins are well-established as playing significant roles in cancer development and progression [34]. Notably, SP1 can generate tissue-specific gene expression programmes with or without tissue-specific transcription factors by modifying binding/transactivation at a specific site, and epigenetic changes can affect the availability of binding sites [35]. By analysing ChIP-seq data for SP1, SP2, and SP5 in the liver cancer cell line HepG2 (downed from ENCODE) [36], we found direct binding of SP1, SP2, and SP5 to the promoters of most SRTs near TSSs, which exhibited strong H3K27ac occupancy and open chromatin accessibility (Fig. 3h and Fig. S3d). Furthermore, GSEA demonstrated a significant association between the SRT signature and SP1 expression (Fig. 3i). We then performed RNA-seq to elucidate the underlying transcriptional programmes affected by SP1. Interestingly, transcripts downregulated upon SP1 knockdown in Huh 7 cells were enriched in the SRT signature (Fig. S3e). Figure 3j depicts the SP1-bound regions, H3K27ac occupancy, and open chromatin accessibility in the promoter region of two HCC-SRTs: CCNE1 and CCNE2. Additionally, knockdown of SP1 resulted in downregulation of these two HCC-SRTs. Collectively, these findings indicate the presence of epigenetic alternations at the genomic location of SRTs in HCC, potentially contributing to the generation of SRTs.

### Landscape of isoform switching events in primary Liver cancer

**Fig. 4:**
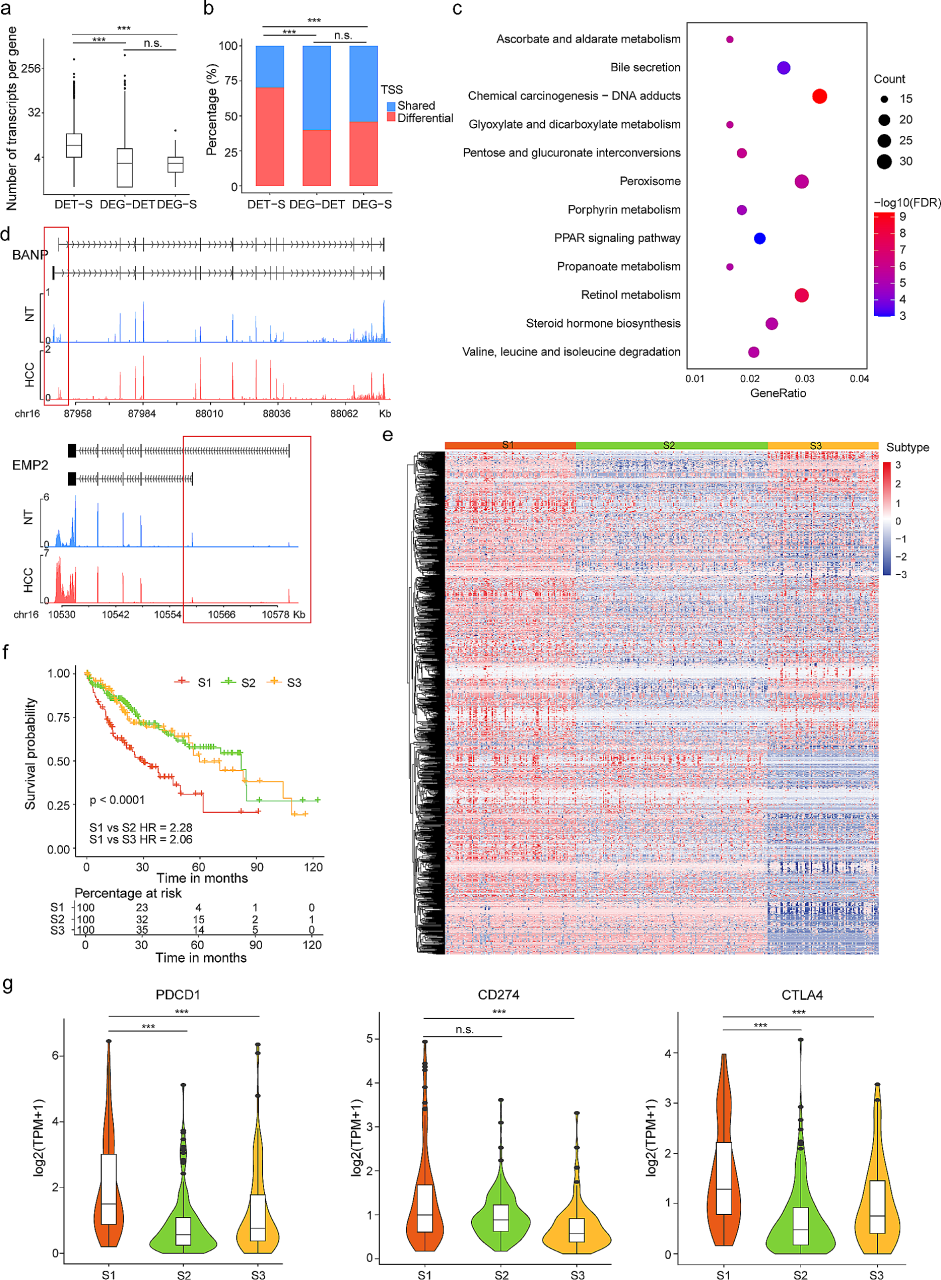
Landscape of isoform switching events in primary liver cancer. **a**. Comparison of the average number of transcripts per gene for each item in Fig. 2c. P-values for the abundance of transcripts per gene in DET-specific and DEG-specific genes were calculated using the Wilcoxon rank-sum test. **b**. Percentage of genes with multiple TSSs for each item. P-values for the enrichment of genes with multiple TSS in DET-specific and DEG-specific genes were calculated using a chi-squared test. **c**. Pathway enrichment analysis for gene associated with isoform switch transcripts. **d**. Structure of the BANP and EMP2 transcripts in HCC and HCC-NT. **e**. K-means clustering of the isoform switch ratio. **f**. Kaplan-Meier curves illustrating overall survival in different isoform switch cluster patterns in the TCGA-LIHC cohort. Statistical significance was determined using Log-rank tests. Hazard ratio (HR) was calculated using the Cox proportional hazards regression model. **g**. Differences in the expression of immune checkpoint inhibitors among different types of isoform switch cluster patterns. ***p < 0.001, n.s., not significant

As mentioned above, the majority of DETs belonged to genes that showed no changes in gene expression levels. To investigate this further, we compared the numbers of transcripts identified in our Iso-Seq data with the TSSs of each gene in the database. The results showed a significantly higher number of transcripts for DET-specific genes than DEGs, and they also exhibited a large number of differential TSSs (Fig. 4a-b). These findings suggest the occurrence of isoform switching events in these DETs despite no gene expression changes. Isoform switching is a prominent feature in cancer, and the alternative usage of transcript isoforms from the same gene can have diverse biological impacts. Consequently, we conducted an analysis to characterize isoform switching events by comparing the expression of specific isoforms in HCC and HCC-NT. In total, 1566 genes exhibited significant isoform switching events (Fig. S4a and Table S4). These genes are primarily associated with signal transduction, and metabolism, and are components of known cancer gene signatures (Fig. 4c-d).

Next, we applied consensus clustering to classify patients into three subtypes based on the ratio of isoform switching (Fig. 4e). In our prognostic analysis of isoform switch patterns, we observed a particularly unfavourable survival outcome in patients classified under Cluster_S1 (Fig. 4f). Additionally, we found that PDCD1 (PD-1) and CTLA4 were significantly upregulated in the Cluster_S1 subtype compared to the other subtypes; CD274 (PD-L1) showed significant upregulation in the Cluster_S1 subtype compared to the Cluster_S3 subtype (Fig. 4g). Moreover, the Cluster_S1 subtype exhibited a positive association with several cancer hallmarks, including cell proliferation, stemness, glycolysis, hypoxia, and inflammation (Fig. S4b). Interestingly, there were no associations between isoform switch subtypes and EMT or angiogenesis.

### Tissue-specific transcript reprogramming is associated with accessible transposable elements and altered TSSs

**Fig. 5:**
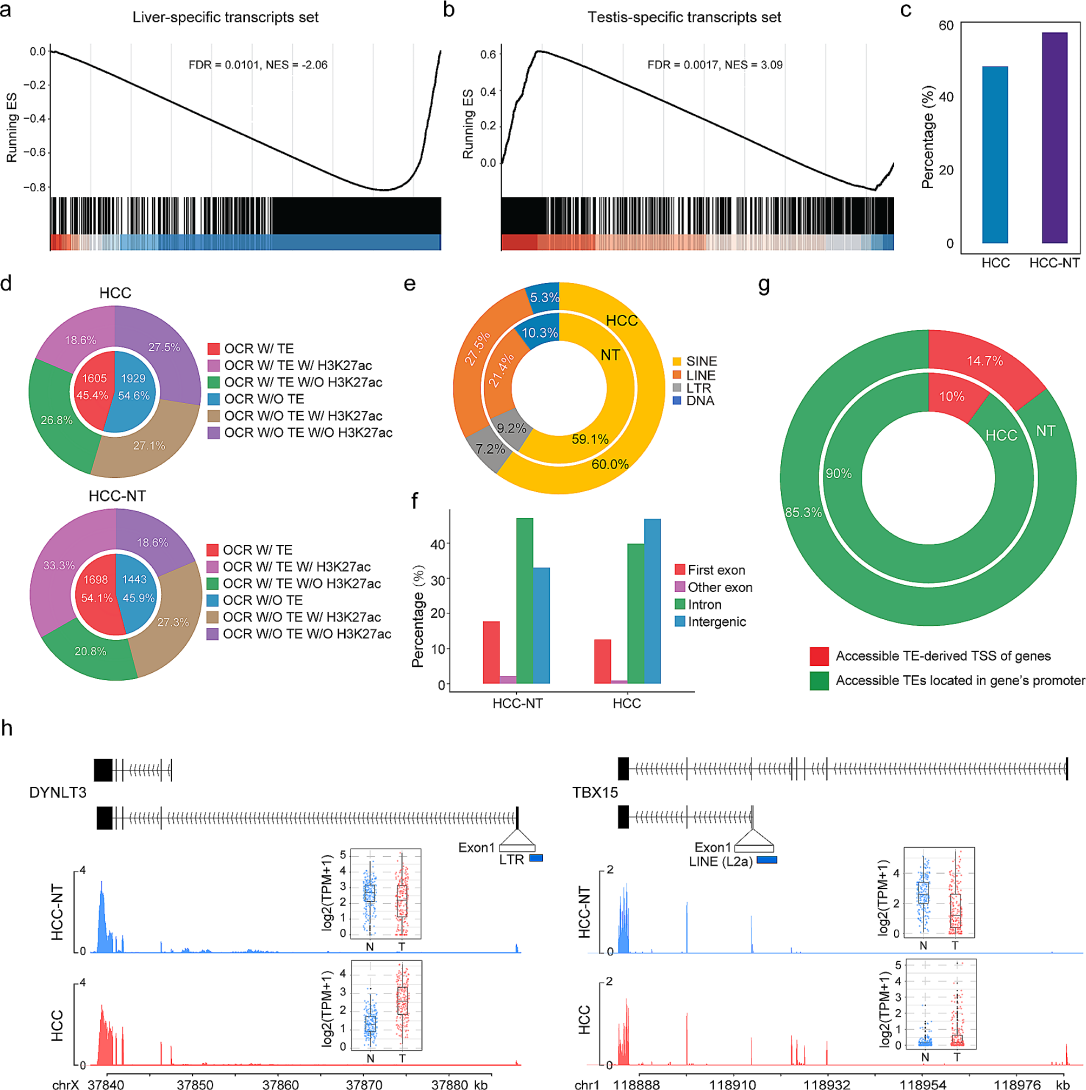
Tissue-specific transcript reprogramming is associated with accessible transposable elements and altered transcription start sites. **a**-**b**. GSEA was performed on liver-specific transcripts and testis-specific transcripts obtained from the GTEx database. The transcripts were ranked based on the log2-fold change of the TPM values between HCC and HCC-NT samples. **c**. The percentage of isoform switching events that involve the presence of TEs. **d**. Identification of H3K27ac modification and ATAC-seq peaks associated with TEs in HCC and HCC-NT groups. **e**. Distribution of TE classes among accessible TEs in HCC and HCC-NT groups. **f**. Enrichment analysis of the genomic distribution of accessible TEs in HCC and HCC-NT. **g**. Distribution of accessible TEs in gene promoters and TSSs derived from TEs in HCC and HCC-NT. **h**. Analysis of H3K27ac occupancy profiles surrounding accessible TSSs derived from TEs in isoform switch transcripts of HCC and HCC-NT

Notably, when comparing the isoform switch profiles of HCC and matched nontumor liver tissues in GTEx normal tissues, GSEA revealed that the significantly downregulated transcripts in HCC were enriched for a liver-specific signature (Fig. 5a). In contrast, the dominantly expressed transcripts in matched nontumor liver tissues are liver specific, while many other tissue-specific transcripts, particularly in the testis, showed upregulation in HCC. This finding suggests that transcript reprogramming resembling embryonic patterning occurs during HCC initiation (Fig. 5b and Fig. S4c).

Transposable elements (TEs) constitute a substantial portion of eukaryotic genomes and play a role in tissue-specific gene expression during development. Approximately 45% of the human genome is derived from different kinds of TEs [37–39]. Our analysis revealed approximately 50–60% of isoform switching events to involve the presence of TEs (Fig. 5c). Interestingly, transcripts with downregulated expression in HCC exhibited even higher levels of TEs. To explore the potential role of TEs in regulating isoform switching in HCC, we analysed ChIP-seq and ATAC-seq data from paired HCC and HCC-NT tissues. We found 45.4% (HCC) to 54.1% (HCC-NT) of TEs annotated within the promoter regions of transcripts to be associated with open chromatin regions (OCRs), with more than half of these accessible TEs overlapping with the active histone modification H3K27ac (Fig. 5d). On average, approximately 60% of the accessible TEs belong to the SINE class, and ~ 24.5% are LINEs. LTR elements accounted for 8.2% of the remaining TEs, and less than 8% belong to the DNA class (Fig. 5e). Approximately 80% of the accessible TEs are located in intragenic or intergenic regions, with some overlapping with exons (Fig. 5f), suggesting their potential role as promoters or enhancers in isoform switching. Moreover, we observed that most of the accessible TEs are located in the promoter regions (0.5 kb upstream of the TSS) of genes (Fig. 5g-h). In particular, approximately 11% of the accessible TEs directly overlap with the TSS of genes, indicating their potential involvement in RNA polymerase II recruitment and altered transcription initiation of downstream genes.

### Long-read transcriptome profiles and characteristics of liver metastases

**Fig. 6:**
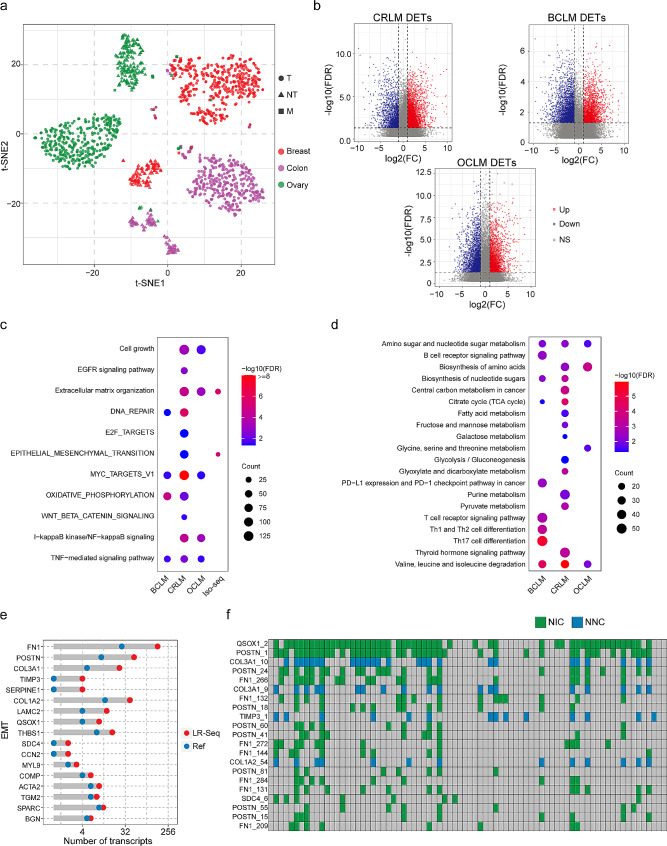
Long-read transcriptome profiles and characteristics of liver metastases. **a**. Expression patterns of well differentiated transcripts, visualized through a t-SNE projection of CRLM, BCLM, OCLM, and primary cancers and corresponding normal samples. **b**. Volcano plot displaying DETs in CRLM, BCLM, and OCLM compared to primary cancer from TCGA. Red and blue dots represent transcripts that were significantly upregulated and downregulated (FDR < 0.05), respectively. **c**. Pathway enrichment analysis of genes associated with upregulated transcripts. **d**. GO analysis of genes associated with downregulated transcripts. **e**. Comparison of the number of novel transcripts to annotated GENCODE transcripts for genes associated with EMT. **f**. Heatmap illustrating the expression of novel transcripts of EMT genes in tissues from individual LM patients

To investigate variations in the metastatic transcriptome, we analysed long-read transcriptomes from primary cancer and liver metastasis samples. Initially, we utilized the Combat method from the R package SVA for batch effect correction and employed t-SNE to visualize the qualitative expression patterns of transcripts across our primary cancer and liver metastasis samples, and primary cancer and nontumor samples from TCGA. The results demonstrated highly tissue-specific transcript expression, effectively segregating normal and cancer samples across different tissues. Interestingly, these transcripts also exhibited distinct separation between primary cancer and metastatic samples, with metastatic samples clustering together to a certain extent, regardless of the type of primary cancer (Fig. 6a).

We then evaluated the extent to which cancer-specific transcript expression is maintained in metastatic lesions (Fig. S5a). Compared with normal tissues from GTEx, the DETs in CRLM, BCLM, and OCLM overlapped based on upregulation and downregulation (Fig. S5b-c). Enrichment analysis of these overlapping DET-associated genes using KEGG and MSigDB cancer hallmarks demonstrated increased transcriptional output for most oncogenic pathways and principal cancer hallmarks compared to normal tissues. Downregulated DET-associated genes were found to be enriched in metabolism and cancer-immune responses (Fig. S5d-e).

Next, we compared the DETs between LM and primary cancer tissues to evaluate metastasis-specific transcript variations (Fig. 6b, Fig. S5f-g, and Table S5). Metastatic tumours exhibit a global increase in cell growth, angiogenesis, and apoptosis. Notably, spliced genes with novel isoforms showed strong associations with extracellular matrix organization and EMT (Fig. 6c-d). We further examined individual EMT genes that displayed a high gain of novel splice isoforms in the liver metastasis transcriptome. In total, 23 genes exhibited a 2.125-fold increase in novel isoforms not present in the reference (GENCODE v.44) (Fig. 6e-f).

We also compared the DEGs between CRLM, BCLM, and OCLM and previously published nonmetastatic colon cancer (COAD), breast cancer (BRCA), and ovarian cancer (OV) cases from TCGA (Fig. S6a). The Venn diagram in Fig. S6b indicates that the DETs encompassed the majority of DEGs. Moreover, we overlapped the upregulated and downregulated DEGs in CRLM, BCLM, and OCLM. As expected, the shared deregulated genes in LM based on isoform expression data included most of the DEGs identified using gene expression data (Fig. S6c-d), highlighting the power of performing differential expression analysis using isoform expression data.

### Metastasis-specific transcripts predict the Metastasis and tissue origin of liver metastases

**Fig. 7:**
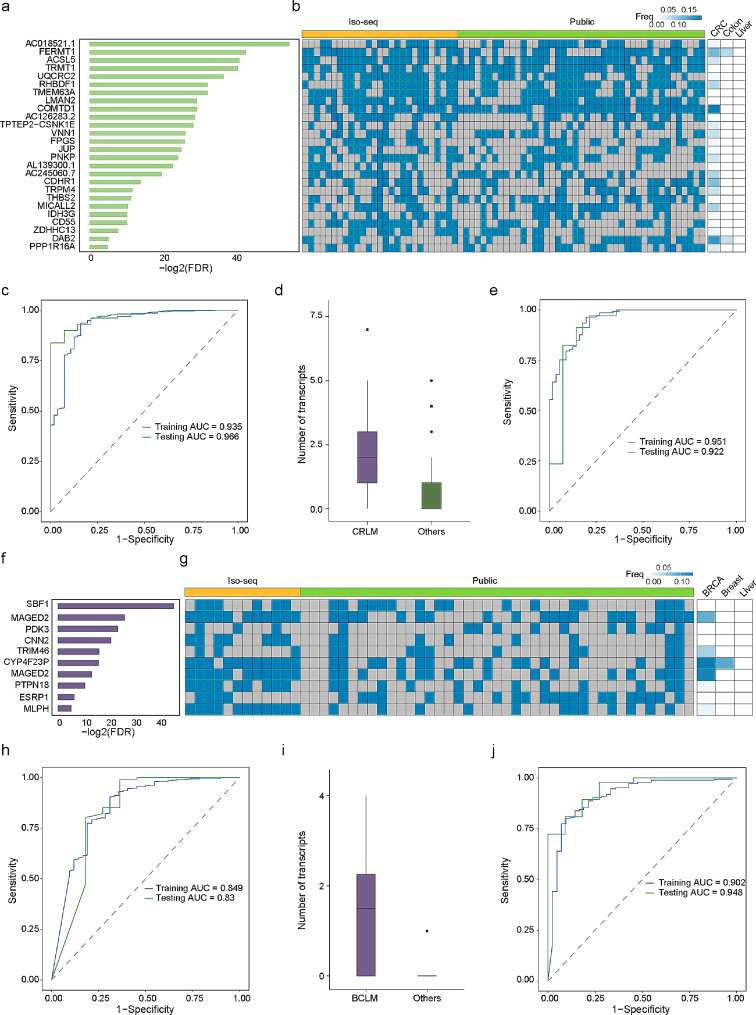
Metastasis-specific transcripts predict the metastasis and tissue origin of liver metastases. **a**. Barplot illustrating the statistical significance of 26 CRLM-SRTs. Metastasis-specific transcripts were defined as transcripts with a fold change greater than 10 between metastatic and nonmetastatic tumours. **b**. Heatmap displaying the expression of the indicated novel transcripts in tissues from individual CRLM patients, including data from Iso-seq and public dataset, as well as TCGA-CRC patients and normal colon and liver tissues. **c**. Area under the curve (AUC) estimation for the CRLM-SRT panel in the training and validation datasets, assessing its ability to discriminate CRLM from CRC. **d**. Boxplot demonstrating the number of detected SRTs by Iso-Seq in CRLM patients compared to LM patients with other tissue origins. **e**. AUC estimation for the CRLM-SRT panel in the training and validation sets, evaluating its effectiveness in identifying the tumour tissue origin of the colon. f. Barplot illustrating the statistical significance of 10 BCLM-SRTs. **g**. Heatmap displaying the expression of the indicated novel transcripts in tissues from individual BCLM patients, including data from Iso-seq and public datasets, as well as TCGA-BRCA patients and normal breast and liver tissues. **h**. AUC estimation for the BCLM-SRT panel in the training and validation sets, assessing its ability to discriminate BCLM from breast cancer. **i**. Boxplot illustrating the number of detected SRTs by Iso-Seq in BCLM patients compared to LM patients with other tissue origins. **j**. AUC estimation for the BCLM-SRT panel in the training and validation sets, evaluating its effectiveness in identifying the tumour tissue origin of the breast

Specific genetic alterations are associated with different tumour types, and analysing genomic features can provide precise and relevant clinical information for disease management. To gain further insight into the impact of metastasis-specific transcripts on metastasis diagnosis, we assessed tumour SRTs in patients with CRLM and BCLM. In the context of CRLM, we identified 26 CRLM-SRTs that exhibited significantly higher expression in CRLM patients than in CRC patients from the TCGA dataset (Fig. 7a). Moreover, these SRTs were barely expressed in other TCGA patients and several normal tissues, including colon, liver, breast, and lung tissues (Fig. 7b and Fig. S7a-b).

To evaluate the diagnostic value of CRLM-SRT expression for liver metastasis in CRC patients, we conducted receiver operating characteristic (ROC) curve analysis to assess the efficacy of the CRLM-SRT panel in discriminating liver metastasis in patients with CRC. By using a random sampling approach, we divided combined samples from the CRLM tissue RNA-seq dataset and the TCGA colon and rectal cancer RNA-seq dataset into a training dataset (80%) and a testing dataset (20%) to construct the CRLM-SRT panel for liver metastasis diagnosis. The results demonstrated that the CRLM-SRT panel had high accuracy in discriminating CRLM from CRC patients (AUC for training set = 0.935; 95% CI, 0.898 to 0.972; AUC for testing dataset = 0.966; 95% CI, 0.935 to 0.997) (Fig. 7c).

Liver cancer of unknown primary (CUP) is the most common subgroup of CUP and has particularly poor prognosis [40]. We observed high expression levels of most CRLM-SRTs in CRLM patients compared to other liver metastasis (LM) patients (Fig. 7d). We further conducted ROC analysis to evaluate the performance of the CRLM-SRT panel and explore its potential diagnostic utility for LM. We divided combined samples from our LM tissue RNA-seq dataset and the LM tissue RNA-seq dataset from the MET500 dataset [26] into a training dataset (80%) and a testing dataset (20%). The CRLM-SRT panel exhibited an overall accuracy of 90.3% in identifying the tumour tissue origin (colon) in liver metastatic tumour samples. The AUCs for the CRLM-SRT panel were 0.951 for the training dataset and 0.922 for the testing dataset (Fig. 7e).

Similarly, we identified 10 BCLM-SRTs specifically expressed in BCLM patients compared to BRCA patients from the TCGA dataset (Fig. 7f-g and Fig. S7c-d). The predicted probability was used to construct a ROC curve, and the AUCs for the BCLM-SRT panel were 0.849 for the training dataset and 0.83 for the testing dataset in discriminating BCLM from breast cancer (Fig. 7h). High expression levels of most BCLM-SRTs were also observed in BCLM patients compared to other LM patients (Fig. 7i). The AUCs for the SRT panel in predicting the tumour tissue origin (breast) in liver metastasis patients were 0.951 for the training dataset and 0.922 for the testing dataset (Fig. 7j).

### Altered transcriptome profiles and characteristics of the liver surrounding liver metastases

**Fig. 8:**
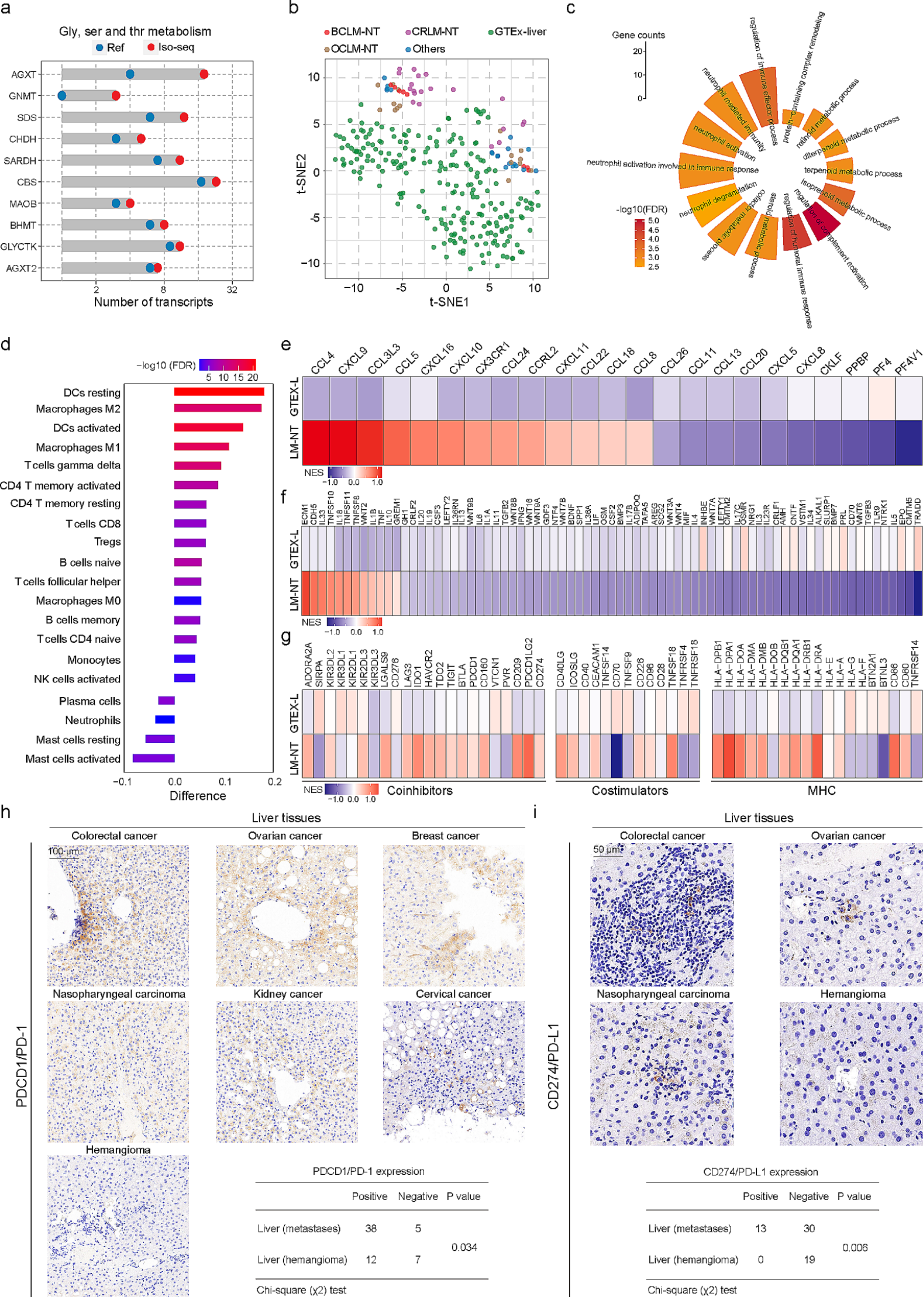
Altered transcriptome profiles and characteristics of the liver in metastatic liver cancer. **a**. Comparison of the number of novel transcripts to annotated GENCODE transcripts for genes related to glycine, serine and threonine metabolism. **b**. Global transcript expression patterns of liver samples depicted through a t-SNE projection, including LM-NT samples and GTEx normal liver samples. **c**. Pathway enrichment analysis of genes associated with DETs in LM-NT samples. **d**. The difference in relative abundance of infiltrating immune cells between LM-NT and GTEx normal liver samples using the CIBERSORT algorithm. A difference > 0 indicates the enrichment of immune cells in LM-NT, and the color of the column represents the statistical significance of the difference. **e**-**f**. Changes in mRNA expression of chemokines, cytokines, and their receptors in LM-NT compared to GTEx normal liver samples. **g**. Change in mRNA expression of major histocompatibility complex (MHC) molecules, costimulators, and coinhibitors in LM-NT compared to GTEx normal liver samples. **h**-**i**. Representative IHC images displaying the expression of PD-1 and PD-L1 in LM-NT and hemangioma liver tissues. Scale bars, 100 μm or 50 μm

To gain insights into the implications of altered liver transcriptomes surrounding liver metastases, we initially analysed the novel transcripts obtained from our Iso-Seq data of nontumor livers (Table S6). Genes were ranked based on their ratio of isoform number gain compared to GENCODE v.44. Genes showing a fold increase of more than one were selected for pathway enrichment analysis. We found spliced genes with novel isoforms to be strongly associated with metabolism (Fig. S8a). Genes associated with glycine, serine and threonine metabolism, which exhibited a high gain of novel splice isoforms, were further investigated in the liver transcriptomes. A total of 10 genes showed a 1.5-fold increase in NIC + NNC isoforms compared to the reference (Fig. 8a). Next, we utilized the Combat method from the R package SVA for batch effect correction, and compared the transcriptomes between the nontumor liver tissues surrounding liver metastases and GTEx normal liver tissues to explore factors that may favour liver organotropism (Fig. 8b). We identified a total of 3380 DETs between these two groups (Fig. S8b and Table S7). Pathway enrichment analysis revealed these DETs to be enriched in immunological and metabolic alterations, particularly functions associated with the immune response, such as regulation of immune effector process, neutrophil activation, complement activation, and humoral immune response (Fig. 8c). Enrichment of metabolic alterations was associated with glucose uptake and central carbon metabolism, including the TCA cycle, glycolysis, hypoxia, and amino acid and fatty acid metabolism. (Fig. S8c).

Furthermore, we analysed the compositions of different immune cells using a reference microenvironment compendium (CIBERSORT) [41]. We observed altered compositions of immune cell types in the liver tissues surrounding liver metastases. Most cell types, including adaptive immune cells and activated/inactivated innate immune cells, were found to be abundant in the liver surrounding the LM, such as resting dendritic cells, M2/M1 macrophages, and T and B cells (Fig. 8d). A significant increase in M2 macrophages and T regulatory cells suggested the role of macrophage activation and immunosuppression in immune escape during liver metastasis. In addition, the liver surrounding LM exhibited higher expression of chemokines, including CCL4 and CCL5, which are known to attract monocytes and CD 8 + T cells (Fig. 8e), as well as highly expressed secreted immunostimulatory and immunoinhibitory cytokines (Fig. 8f). Expression of immune checkpoint molecules following immune stimulation is a potentially important intrinsic immune escape mechanism. We found that the liver surrounding LM had higher expression of costimulatory molecules (most with *P* < 0.05) and immune checkpoint molecules (most with *P* < 0.05) than the GTEx normal liver (Fig. 8g). Moreover, we investigated the relationship among immune cells, cytolytic activity (CYT), and expression of immune checkpoint molecules. The results showed a positive correlation between CYT and expression of most checkpoint molecules (Fig. S8d). Deregulated expression of immune checkpoint molecules, such as the immune checkpoint inhibitors (ICIs) PDCD1 (PD-1) and CD274 (PD-L1), in adjacent nontumor liver tissues from LM patients, including colorectal cancer, ovarian cancer, breast cancer, nasopharyngeal carcinoma, kidney cancer, and cervical cancer liver metastases, was validated by immunohistochemistry (IHC) staining. Consistent with the ICI expression pattern, PD-1- and PD-L1-positive cells exhibited a significant increase in liver tissues surrounding LM (Fig. 8h-i).

## Discussion

A large number of previously undocumented transcripts exhibit critical cellular functions, and their aberrant expression might contribute to carcinogenesis [9–11], which suggests that long-read sequencing technologies provide a good understanding of cancer transcripts. In this study, 37,888 isoforms were uncovered in HCC, 41.2% of which are novel when compared with the reference transcriptome. Several transcripts and transcriptional outputs were increased for most oncogenic signatures, indicating a global shift towards a cancer-related transcriptional programme. Emerging evidence has demonstrated the importance of determining the SRTs of genes in particular physiological and pathological conditions [42]. Our previous studies analysed normal and cancer RNA-seq samples to identify SRTs exclusively expressed in cancer samples and to construct an SRT database across various cancer and tissue types [43]. In the present study, we used long-read sequencing to provide more accurate and complete transcriptome-enabling analyses at SRT resolution and highlight their potential clinical utility in HCC prognosis. Various regulatory properties contribute to the generation of SRT, such as activation of alternative promoters, alternative splicing, and/or polyadenylation. Based on the present study, we suggest that Sp family proteins might mediate promoter changes to activate a set of SRTs in HCC. Posttranscriptional modifications are known to be extensively regulated by RNA-binding proteins (RBPs), which can modulate expression levels of transcripts. To gain further insights, we analysed the correlation between expression of SRTs and individual RBPs, and found that expression of certain RBPs exhibited a significant correlation with SRTs (data not shown). This suggests that RBPs may also contribute to the generation of SRTs. To comprehensively capture the complete landscape of SRT generation, future studies should explore additional layers of regulatory control.

Isoform switching events with predicted functional consequences are common in many cancers. Our group and others revealed that some splicing switches contribute essentially to hepatic carcinogenesis and could serve as promising therapeutic targets for HCC [42, 44, 45]. However, comprehensive characterization of the switching events in HCC using long-read sequencing is lacking. Herein, we describe large numbers of events linked to HCC, and patients with specific isoform switch patterns had worse prognosis. Notably, we discovered reprogrammed tissue-specific transcription, such as simultaneous loss of liver-specific and gain of other tissue-specific transcription programmes, in hepatocarcinogenesis. The intact transcriptional elements of TEs can create novel TSSs to initiate transcription in the host genome [46, 47]. Our results highlighted the contribution of TEs to tissue-specific transcriptional reprogramming during carcinogenesis, especially to creating novel tissue-specific transcript expression patterns by acting as alternative TSSs or potential enhancers.

Progress in the past few decades has greatly enhanced our understanding of the molecular and cellular nature of both the ‘‘seed’’ and ‘‘soil’’ [48]. It has long been observed that most cancers show an organ-specific pattern of metastases, and the liver is one of the favoured distant metastatic sites for solid tumours [49]. In this study, we investigated metastasis-associated transcriptome variations to explain how metastatic cancer cells with original tissue specificity adapt to the environment of the liver and colonize it. We confirmed that several differentially expressed transcript-associated genes were enriched in metastatic oncogenic signatures. Although several spliced isoforms of these genes have been previously identified, current annotations widely used for transcriptome analysis do not contain the level of complexity revealed by our Iso-Seq analysis. Targeting these specific transcripts might be counterproductive, and inhibiting signalling pathways might be a promising therapeutic strategy. Furthermore, the current study revealed metastasis-specific RNA transcripts in liver metastatic tumours based on the type of primary cancer, which can predict the metastasis potential in individual patients with CRC and breast cancer. Most importantly, expression of these specific transcripts achieved a precise prediction of the tissue origin in liver metastases with an overall accuracy of 81.4%, suggesting that metastasis-specific RNA transcripts can serve as a useful tool for accurately indicating the metastasis potential of primary tumours and clearly identifying the tissue of origin for liver tumours.

Previous studies have reported that the tumour microenvironment (TME) of liver metastasis harbours a highly immunosuppressive phenotype and reprogrammed metabolism, induces a systemic loss of antigen-specific T lymphocytes and gains particular benefits in the metabolically active liver [50, 51]. It remains largely unknown how cancer cells modify the environment of the healthy liver for the homing and hosting of metastatic cells. In this study, we found that adjacent paracancerous liver tissues are abnormal and represent an immunosuppressive microenvironment, which indicates that activation of the chemokine and cytokine signalling pathways, combined with immune evasion and metabolic reprogramming, may play an important role in liver metastasis. Interestingly, patients with non-small cell lung cancer (NSCLC) and liver metastatis treated with nivolumab (anti-PD1 antibody) showed improved overall survival and progression-free survival [52]. In the present study, we found expression of PD-1 and PD-L1 to be upregulated in nontumor liver tissues from a broad range of cancer patients with liver metastasis, which might be a basis for immunotherapy in patients with metastatic liver disease. With more research into the molecular underpinnings of different tumour types, immune checkpoint inhibitors will hopefully continue to be introduced into the clinical setting to treat patients with liver metastasis.

## Conclusions

We comprehensively surveyed the full-length transcriptome landscapes of primary and metastatic liver cancers at transcript resolution. SRTs are frequently expressed, and isoform switching events often occur in HCC with clinical implications and immunological and metabolic alterations to help cancer cells metastasize to the liver. Our findings underscore the significance of exploring the full-length transcriptome profile, which remains an underexplored area of research, and has the potential to provide novel biological insights and biomarkers. The identification of metastasis-specific transcripts that can predict metastatic risk and determine the primary sites of CUP in LM patients holds promise for improving clinical care and patient outcomes.

### Electronic supplementary material

Below is the link to the electronic supplementary material.

Supplementary Material 1

Supplementary Material 2

Supplementary Material 3

Supplementary Material 4

Supplementary Material 5

Supplementary Material 6

Supplementary Material 7

Supplementary Material 8

## Data Availability

The raw data of transcriptome and Iso-seq reported in this paper can be accessed from the Genome Sequence Archive for Human (https://ngdc.cncb.ac.cn/gsa-human/), using the accession number (HRA003557). All data generated or analyzed during this study are included in this published article and its supplementary information files.

## Abbreviations

HCC: Hepatocellular carcinoma
CRC: Colorectal cancer
EMT: Epithelial to mesenchymal transition
Iso-Seq: Isoform-sequencing
PT: Primary tumour
LM: Liver metastases
NT: Nontumor
CRLM: Colorectal cancer liver metastases
BCLM: Breast cancer liver metastases
OCLM: Ovarian cancer liver metastases
FSM: Full-splice match
ISM: Incomplete-splice match
NIC: Novel in catalogue
NNC: Novel not in catalog
DET: Differentially expressed transcript
DEG: Differentially expressed gene
SRT: Specific RNA transcript
ChIP-seq: Chromatin immunoprecipitation sequencing
ATAC: Assay for transposase-accessible chromatin
TSS: Transcription start site
GSEA: Gene set enrichment analysis
TE: Transposable element
COAD: Colon Cancer
BRCA: Breast cancer
OV: Ovarian cancer
ROC: Receiver operating characteristic
CUP: Cancer of unknown primary
