# Supplementary material for: Long-read transcriptome landscapes of primary and metastatic liver cancers at transcript resolution
Source: Biomark Res. 2024 Jan 8;12:4. doi: 10.1186/s40364-023-00554-w (PMC10773130; doi:10.1186/s40364-023-00554-w)
Supplement: Supplementary file 8 — Supplementary Material 8 [file 40364_2023_554_MOESM8_ESM.docx]

**Supporting Information**

**Long-read Transcriptome Landscapes of Primary and Metastatic Liver Cancers at Transcript Resolution**

Zhiao Chen^1,2,3,†^, Qili Shi^1,†^, Yiming Zhao^4,†^, Midie Xu^5^, Yizhe Liu^1^, Xinrong Li^1^, Li Liu^1^, Menghong Sun^5^, Xiaohua Wu^6^, Zhimin Shao^2,7^, Ye Xu^8,*^, Lu Wang^4,*^, Xianghuo He^1,2,3,*，^

^1^Fudan University Shanghai Cancer Center and Institutes of Biomedical Sciences, Shanghai Medical College, Fudan University, Shanghai 200032, China

^2^Key Laboratory of Breast Cancer in Shanghai, Fudan University Shanghai Cancer Center, Fudan University, Shanghai 200032, China.

^3^Shanghai Key Laboratory of Radiation Oncology, Fudan University Shanghai Cancer Center, Fudan University, Shanghai, 200032, China

^4^Department of Hepatic Surgery, Fudan University Shanghai Cancer Center, Fudan University, Shanghai, 200032, China

^5^Department of Pathology, biobank, Fudan University Shanghai Cancer Center, Shanghai, China.

^6^Department of Gynecologic Oncology, Fudan University Shanghai Cancer Center, Fudan University, Shanghai, 200032, China

^7^Department of Breast Surgery, Fudan University Shanghai Cancer Center, Fudan University, Shanghai, 200032, China

^8^Department of Colorectal Surgery, Fudan University Shanghai Cancer Center, Shanghai, 200032, China.

^†^Zhiao Chen, Qili Shi, and Yiming Zhao contributed equally to this article.

**^*^Corresponding Authors:**

Xianghuo He, E-mail: xhhe@fudan.edu.cn, Fudan University Shanghai Cancer Center and Institutes of Biomedical Sciences; Shanghai Medical College, Fudan University, 302 Rm., 7# Bldg., 270 Dong An Road, Shanghai 200032, China. Tel: 86-21-34777329; Fax: 86-21-64172585. Or Lu Wang, E-mail: wangluzl@fudan.edu.cn, Department of Hepatic Surgery, Fudan University Shanghai Cancer Center, Fudan University, Shanghai, 200032, China. Or Ye Xu, E-mail: xuye021@163.com, Department of Colorectal Surgery, Fudan University Shanghai Cancer Center, Shanghai, 200032, China.

**Supplementary Materials and Methods**

**Calculation of microenvironment cell abundance**

The gene signatures of LM22 (22 types of immune cells) were obtained from CIBERSORT[1]. Single-sample gene set enrichment analysis (ssGSEA) [2] was performed to calculate the relative level of each immune cell by the transcriptome of samples.

**Cell culture**

Huh-7 (ATCC, ATCC Number: RCB1366; RRID: CVCL_0336, 2019) was cultured in Dulbecco’s modified Eagle’s medium (DMEM) (Invitrogen, Carlsbad, CA, USA) containing 10% fetal bovine serum (FBS) (HyClone, Logan, UT, USA) and antibiotics (penicillin and streptomycin, Invitrogen) at 37 °C in 5% CO2. Cells were assessed for mycoplasma monthly via the qPCR analysis using specific primers for detecting mycoplasma. All these cells were recently authenticated by STR analysis.

**Oligonucleotide transfection**

SiRNAs or negative control siRNAs (siNCs) were synthesized by RiboBio (RiboBio Biotechnology, Guangzhou, China). Specific siRNA sequences targeting SP1 are as follows (5’-3’): siSP1-1, UGGGAAUUAUGAACUUUACUACC, siSP1-2, GCCUAAUAUUCAGUAUCAAGUAA. Approximately 1-2 × 10^5^ cells were seeded into six-well plates and grown for one day. At 30-40% confluence, cells were transfected with 5 μl of siRNA or siNC (20 μM) using Lipofectamine RNAiMAX (Invitrogen). After 36 h, cells were harvested for further experiments. For RNA-seq, RNA was extracted after 24 h.

**ChIP-seq library preparation and sequencing**

ChIP-seq for HCC and matched non-tumor liver tissues were performed followed the previously published protocol [3]. Briefly, fresh liver cancer or normal tissues were transferred to a clean 1.5-mL tube containing 250 μL of ice-cold PBS and homogenized to yield chunks 0.5 mm^3^. The tissues were cross-linked for 15 min with 1% formaldehyde, followed by the addition of 0.15M glycine to terminate crosslinking. Then washed and gathered the tissues into new tubes. Chromatin was digested into DNA fragments using 0.5 μL Micrococcal Nuclease. The complex of protein and DNA was extracted and resuspended in SimpleChIP Chromatin IP buffers (Cell Signaling Technology, Danvers, Massachusetts, USA), and was incubated with magnetic beads (ThermoFisher Scientific, Shanghai, China) conjugated with 1 μg of the antibody against H3K27ac (Active motif, Shanghai, China, RRID: AB_2561016). The target DNA were washed and eluted using MinElute Spin Columns (Qiagen, Hilden, Germany) for DNA sequencing.

ChIP-sequencing libraries were prepared by using the NEBNext Ultra DNA Library Prep Kit for Illumina (NEB Cat. No. E7370) according to the manufacturer’s instructions. After barcoding, pooled DNA was sequenced (HiSeq 1500, Illumina) to achieve a minimum of 2 × 10^7^ aligned reads per sample. For ChIP-seq analyses, 150-bp paired-end reads were aligned to the reference human genome using Bowtie 2 [4] with standard alignment parameters. PCR duplicates were marked with the Picard “Mark Duplicates” utility and removed from further analysis. Bam files were converted to BigWig files using deepTools [5]. The peak distribution along genomic regions of genes of interest were visualized with IGV.

**ATAC-seq**

Fresh liver cancer or non-tumor liver tissues were cut into 1-2 mm pieces and digested using collagenase. Grinding and counting to isolate 50000 living cells. Transpose, purify and PCR steps were performed as previously described [6]. All deep sequencing was performed on the Illumina HiSeq Xten-PE150 or Illumina HiSeq 2500 platform provided by GENEWIZ (GENEWIZ Suzhou, China). The following tools and versions were used for ATAC-seq data analysis: Trimmomatic [7], SAMtools [8], Picard, and Bowtie2 [4]. First, Nextera adapter sequences were trimmed from the reads by using Trimmomatic. These reads were aligned to a reference genome using Bowtie2 with standard parameters. Picard was then used to remove duplicate reads. These deduplicated reads were then filtered to retain high-quality (MAPQ ≥ 30), non-mitochondrial chromosome, non-Y chromosome, and properly paired (SAMtools flag 0 × 2) reads.

**Human Tissue microarray and immunohistochemistry**

Core samples were obtained from representative regions of each tumor based on H&E staining. Duplicate 1-mm cores were taken from different areas of the same tissue block for each case (intratumoral tissue and peritumoral tissue). Serial sections (4-μm-thick) were placed on slides coated with 3-aminopropyltriethoxysilane. The immunohistochemistry analysis was carried out as described previously. The primary mAbs used were rabbit anti-human PD-1 (1:200, Cell Signaling Technology, Danvers, MA, USA, RRID: AB_2798734) and rabbit anti-human PD-L1 (1:100, Cell Signaling Technology, Danvers, MA, USA, RRID: AB_2728833). The immunostaining intensities was scored semi-quantitatively. All samples were anonymously and independently scored by two investigators. In cases of disagreement, the slides were re-examined, and a consensus was reached by the observers.

**Supplementary Figures**


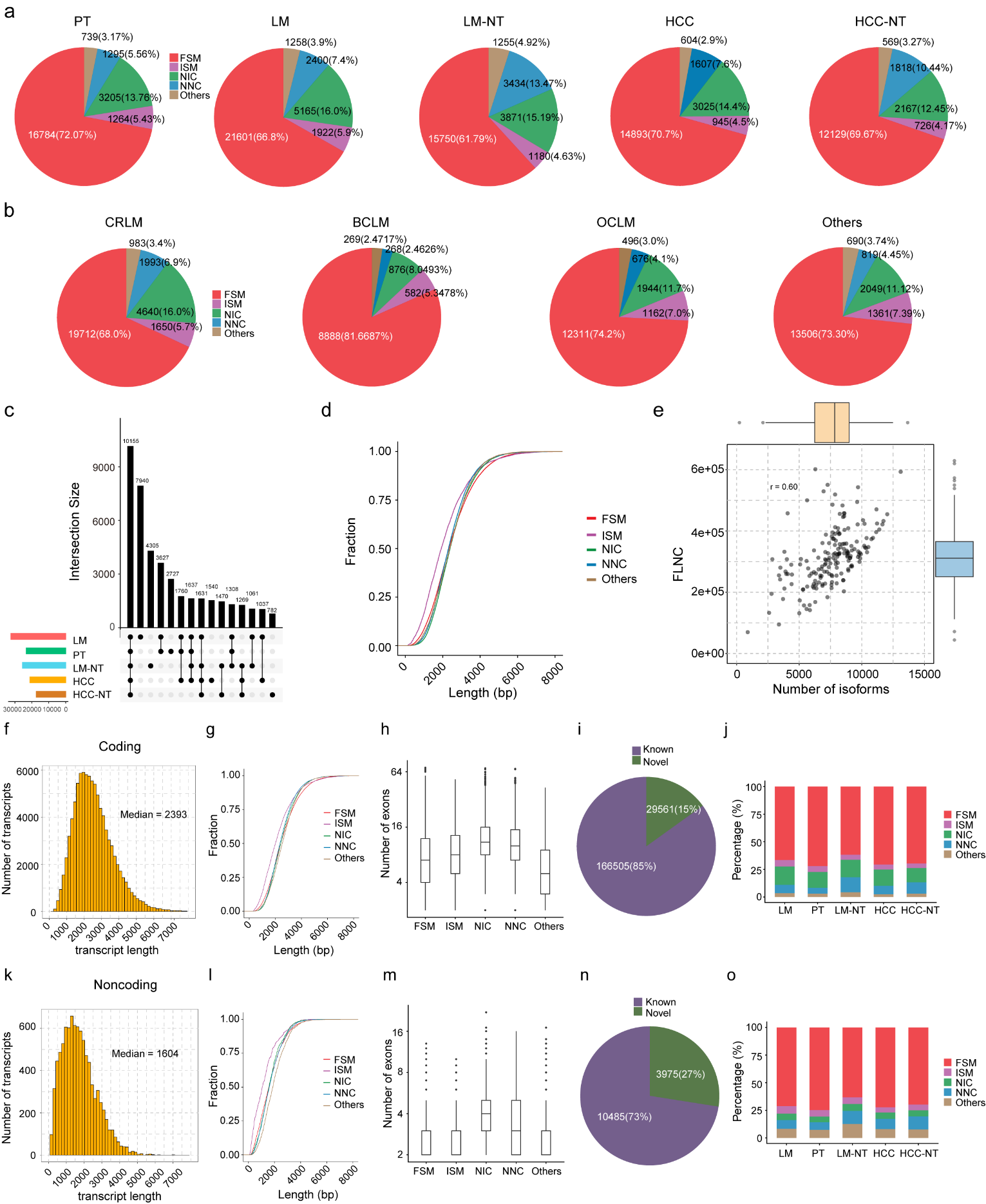
**Fig. S1**

**Fig. S1 Landscape of long-read transcriptomes in primary and metastatic liver cancers**

a. The percent and number of distinct isoforms in each category are indicated in primary and liver metastatic tissues and matched non-tumor tissues. The "LM" group represents the combined transcript list from CRLM, BCLM, OCLM, and others. b. The percent and number of distinct isoforms in each category are indicated in different type of liver metastatic tissues. c. Upset plot of shows isoform interactions among five groups of samples. d. Characteristics of novel and known transcripts. e. Correlation between number of detected isoforms and sequencing FLNC reads. f-h. Characteristics of novel (NIC and NNC) and known (FSM and ISM) coding transcripts. i. Proportion annotation and unannotated junctions in coding isoforms. j. The percent and number of distinct coding transcripts in each category are indicated in primary and liver metastatic tissues and matched non-tumor tissues. k-m. Characteristics of novel (NIC and NNC) and known (FSM and ISM) noncoding transcripts. n. Proportion annotation and unannotated junctions in noncoding isoforms. o. The percent and number of distinct noncoding transcripts in each category are indicated in primary and liver metastatic tissues and matched non-tumor tissues.


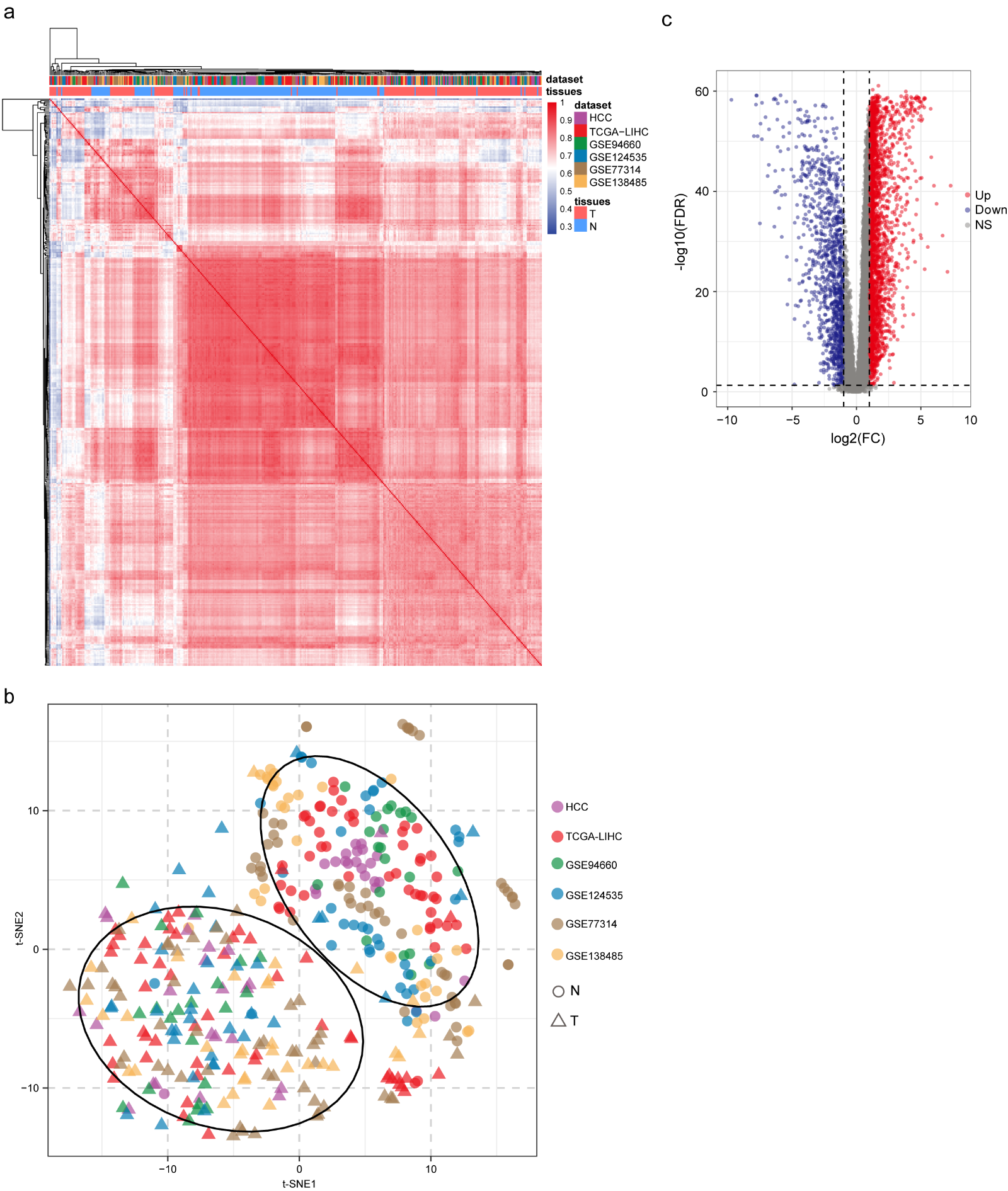
**Fig. S2**

**Fig. S2 Profiling of significant differentially expressed RNA transcripts between primary HCC and matched non-tumor liver tissues**

a. Unsupervised clustering of patient samples (HCC and HCC-NT from various datasets) based on the expression profiles of the transcript. b. Global transcript expression patterns of HCC and HCC-NT samples from various datasets as illustrated by a t-distributed stochastic neighbor embedding (t-SNE) projection. The position of samples within the plot reflects the relative similarity in the expression of transcripts. Samples are color-coded on the basis of their assigned analysis cohort. T, tumor samples; N, non-tumor liver tissues. c. Volcano plot of DEGs in HCC and HCC-NT. Red and blue dots represent transcripts that were significantly upregulated and downregulated (FDR < 0.05), respectively.


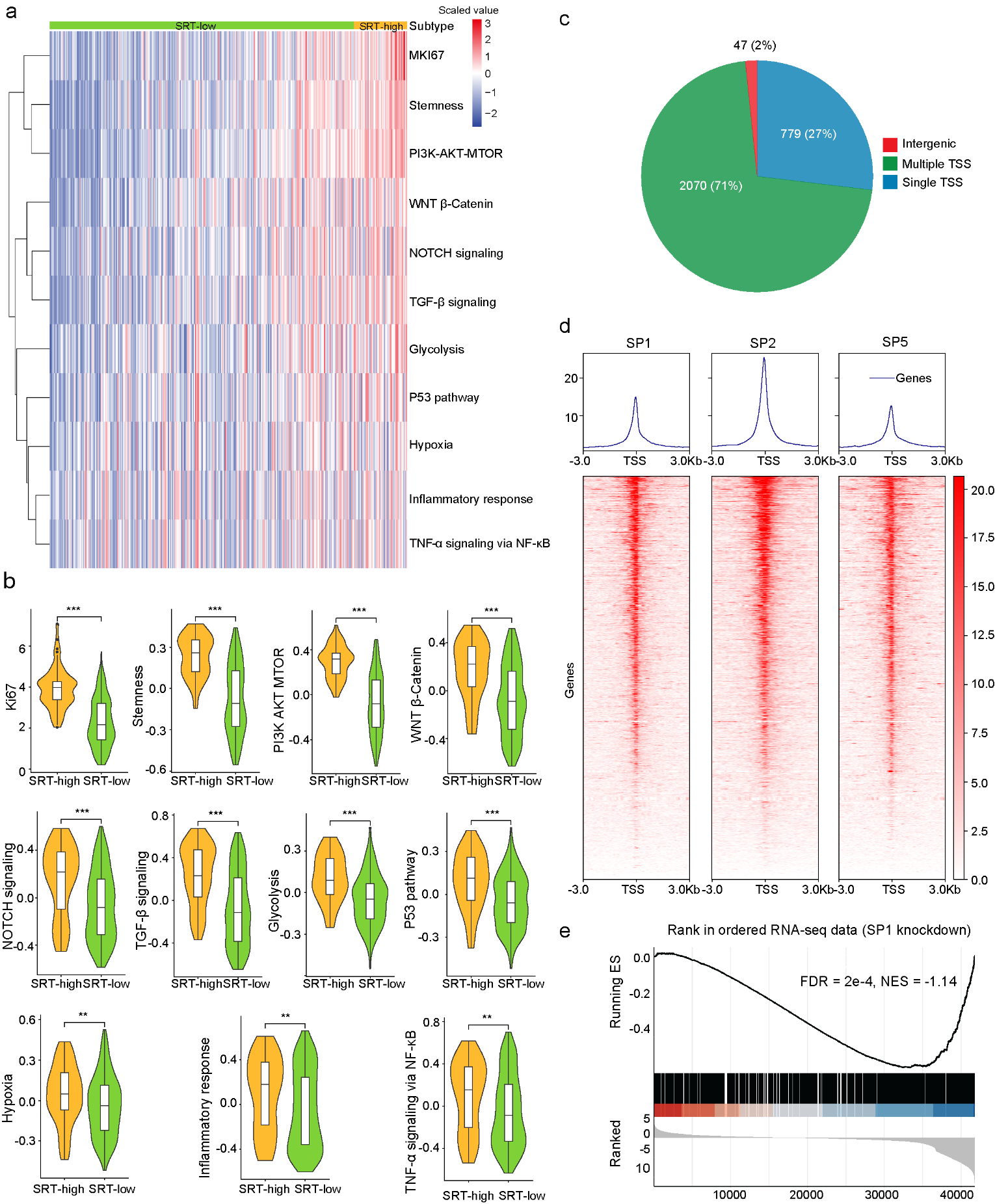
**Fig. S3**

**Fig. S3 Clinical significance and expression control of the SRTs in primary liver cancer**

a. Heatmap visualizing the GSVA enrichment analysis shows the activation states of biological pathways in SRT-high group. b. Differences in the activities of cancer hallmarks between the SRT-high and -low groups. Statistical differences between two groups were determine using the Wilcoxon rank-sum test. c. The pie chart shows the distribution of gene isoforms and intergenic HCC-SRTs. d. Enrichment of ChIP-seq peaks for SP1 and SP2 within 3 kb from the TSSs of HCC SRTs in HCC. e. GSEA of HCC SRTs is shown. Transcripts are ranked by the log2-fold change of the TPM values in Huh 7 siNC and siSP1 cells. The NES and FDR are shown. ***p* < 0.01, ****p* < 0.001.


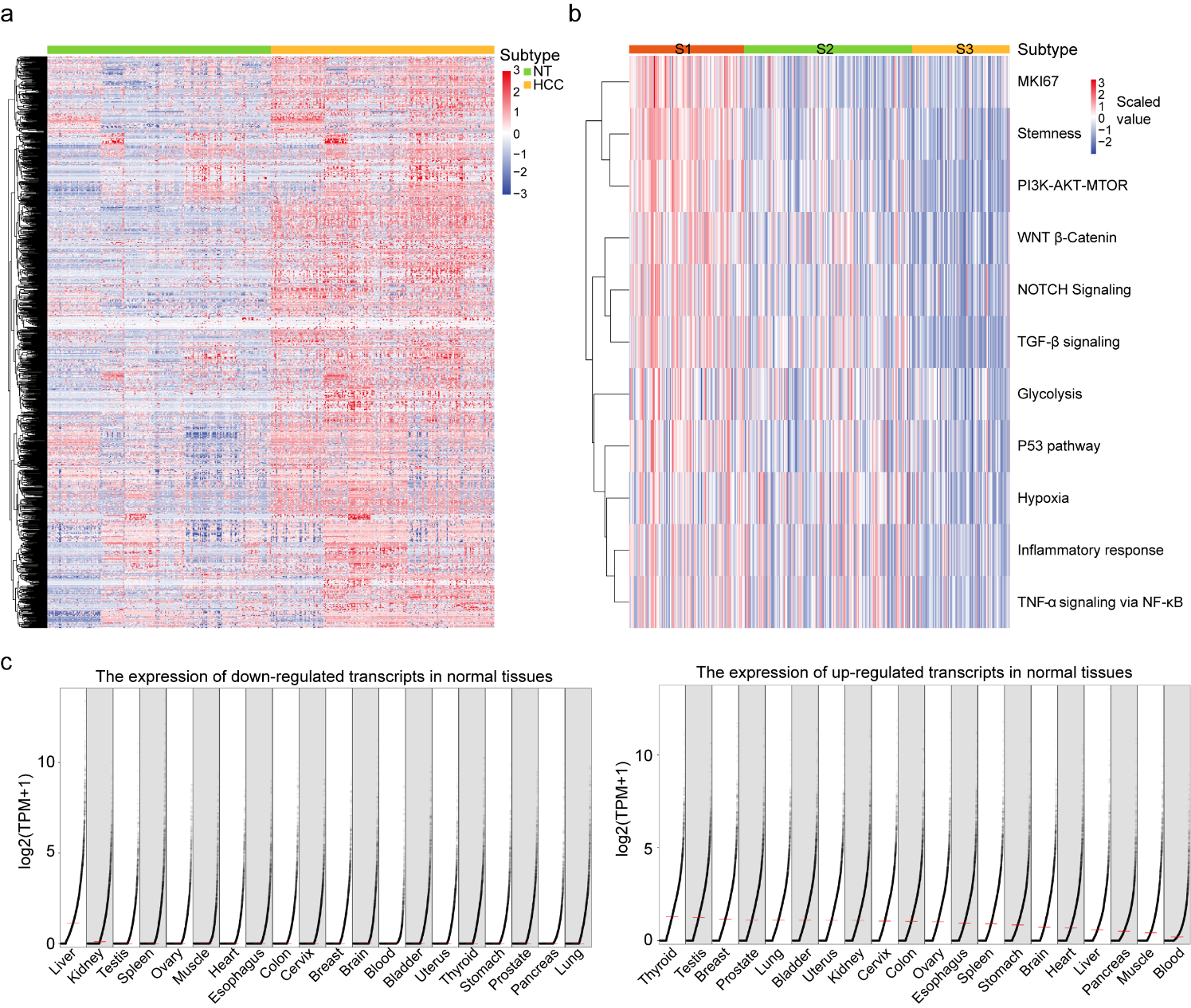
**Fig. S4**

**Fig. S4. Landscape of isoform switching events in primary liver cancer**

a. Heatmap shows the expression levels of isoform switching transcripts in HCC and HCC-NT. b. Heatmap visualizing the GSVA enrichment analysis shows the activation states of biological pathways in isoform switch cluster patterns. c. The expression level of isoform switch transcripts from HCC among various normal tissues.


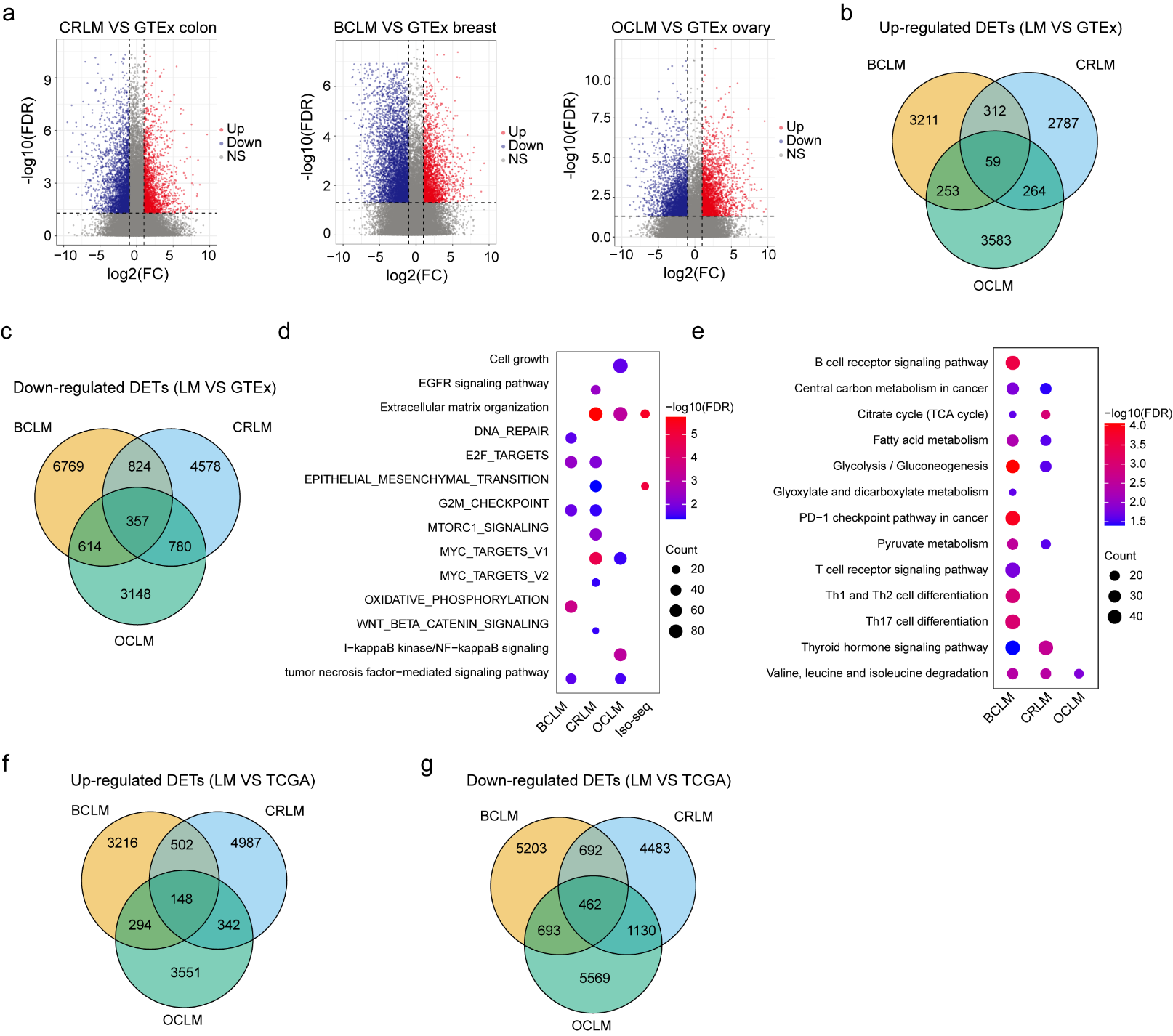
**Fig. S5**

**Fig. S5 DETs in CRLM, BCLM, and OCLM compared to the corresponding normal tissues from GTEx**

a. Volcano plot of DETs in CRLM, BCLM, and OCLM compared to the corresponding normal tissues from GTEx. Red and blue dots represent transcripts that were significantly upregulated and downregulated (FDR < 0.05), respectively. b-c. Venn diagram of overlapping upregulated and downregulated DETs among CRLM, BCLM, and OCLM. d. Pathway enrichment analysis of upregulated transcripts. e. GO analysis of downregulated transcripts. f. Venn diagram of overlapping upregulated DETs among CRLM, BCLM, and OCLM. g. Venn diagram of overlapping downregulated DETs among CRLM, BCLM, and OCLM.


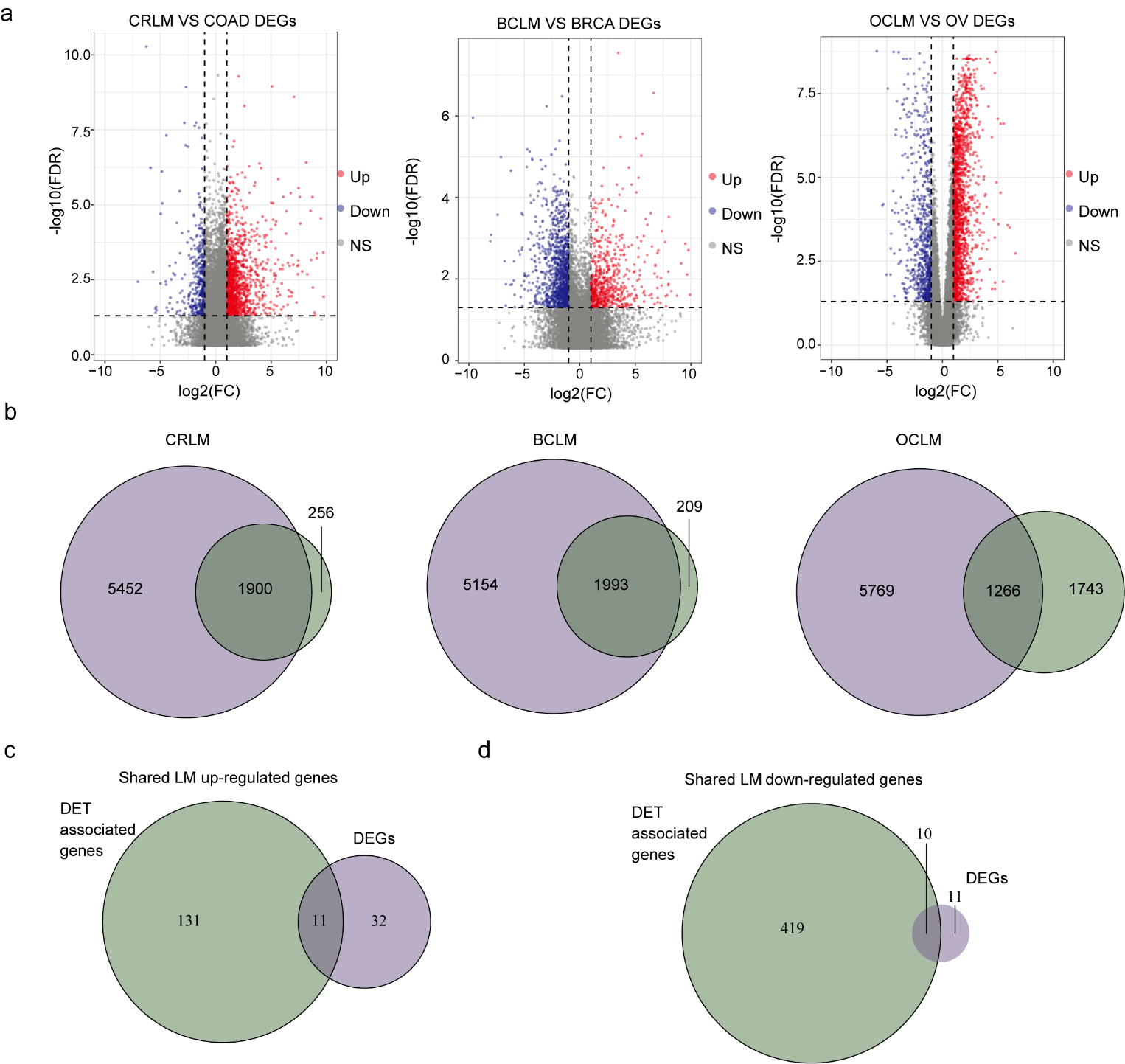
**Fig. S6**

**Fig. S6 DETs and DEGs in CRLM, BCLM, and OCLM compared to the corresponding primary cancer from TCGA data set**

a. Volcano plot of DEGs in CRLM, BCLM, and OCLM compared to the primary cancer from TCGA. Red and blue dots represent transcripts that were significantly up- and down-regulated (FDR < 0.05), respectively. b. Venn diagram of overlapping DETs and DEGs in CRLM, BCLM, and OCLM, respectively. c. Venn diagram of overlapping shared LM up-regulated genes (CRLM, BCLM, and OCLM) from DETs and DEGs. d. Venn diagram of overlapping shared LM down-regulated genes (CRLM, BCLM, and OCLM) from DETs and DEGs.


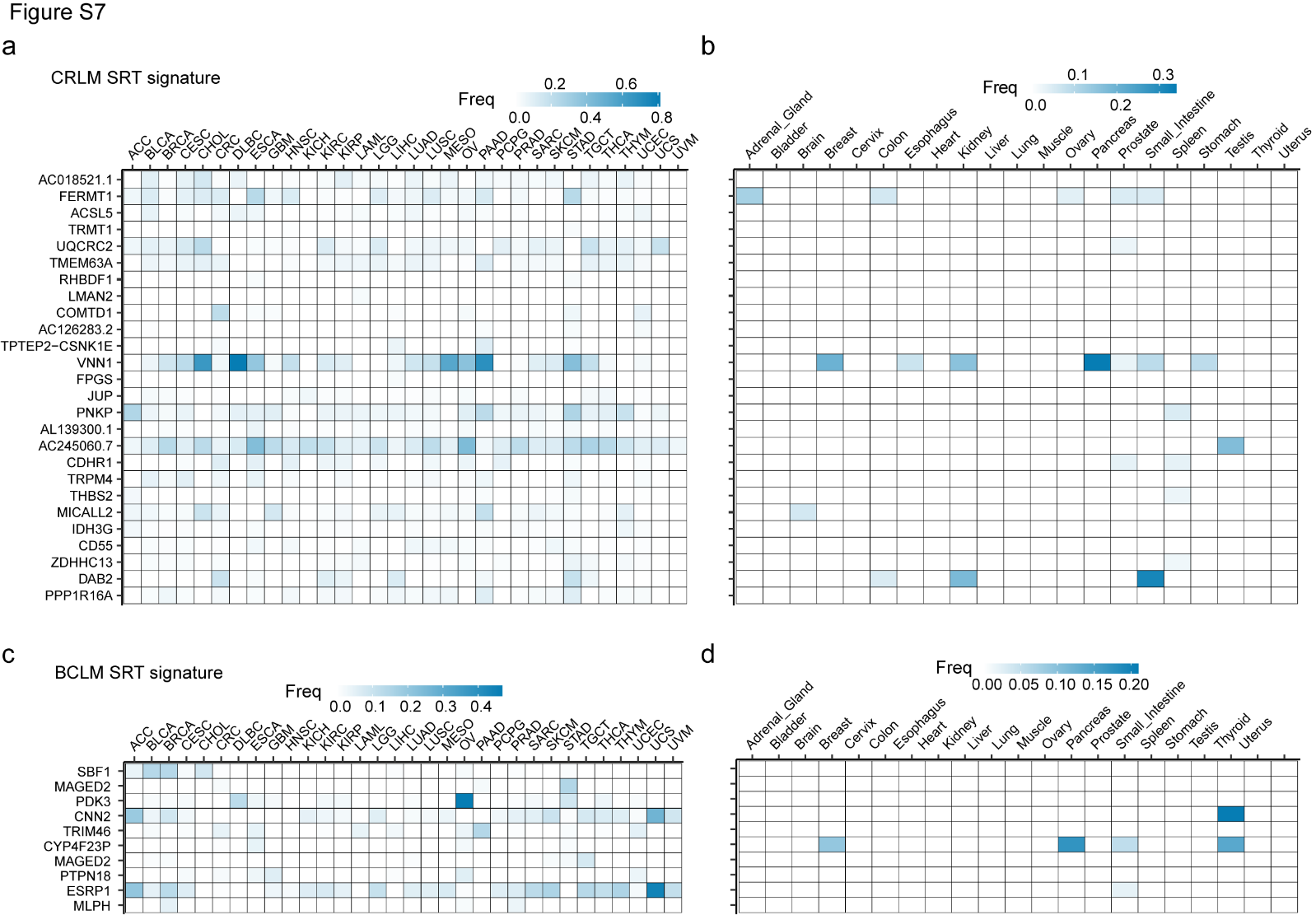
**Fig. S7**

**Fig. S7 Metastasis-specific transcripts predict the metastasis and tissue origin of liver metastases**

a-b. Heatmap shows the expression of indicated CRLM-SRTs in tissues from the individual TCGA patients and normal tissues. c-d. Heatmap shows the expression of indicated BCLM-SRTs in tissues from the individual TCGA patients and normal tissues.


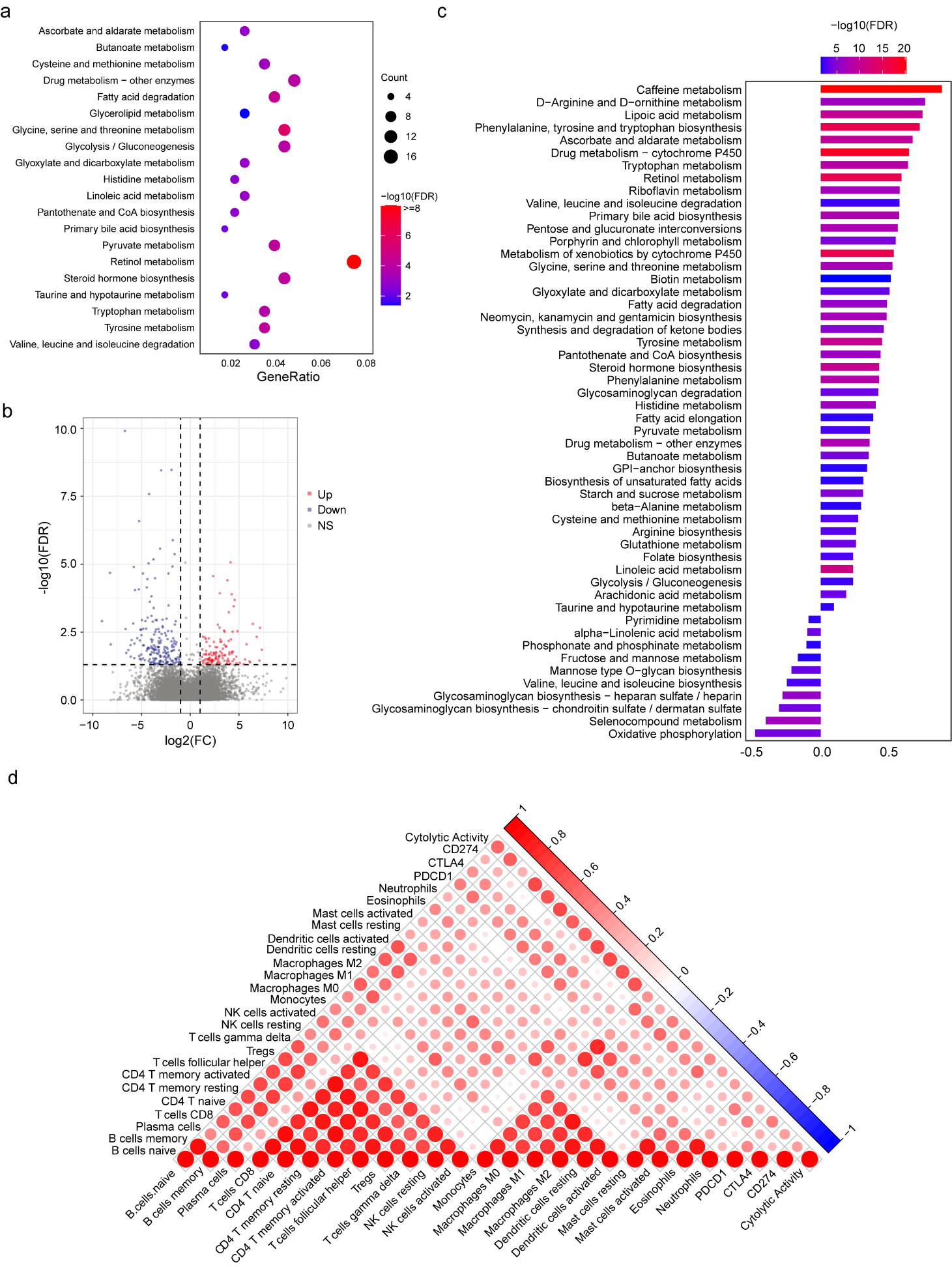
**Fig. S8**

**Fig. S8. Altered transcriptome profiles and characteristics of the liver in metastatic liver cancer**

a. Pathways significantly enriched for genes with novel isoforms detected by Iso-Seq in non-tumor liver tissues from LM patients. b. Volcano plot of DETs in LM-NT compared to GTEx normal liver samples. Red and blue dots represent transcripts that were significantly upregulated and downregulated (FDR < 0.01), respectively. c. The difference in the relative activity of metabolism between LM-NT compared and GTEx normal liver samples. d. Correlations between tumor immunogenicity indicators, immune infiltration, and expression of immune checkpoint molecules.

1. Newman AM, Liu CL, Green MR, Gentles AJ, Feng W, Xu Y, Hoang CD, Diehn M, Alizadeh AA: **Robust enumeration of cell subsets from tissue expression profiles**. *Nat Methods* 2015, **12**(5):453-457.

2. Hanzelmann S, Castelo R, Guinney J: **GSVA: gene set variation analysis for microarray and RNA-seq data**. *BMC Bioinformatics* 2013, **14**:7.

3. Cotney JL, Noonan JP: **Chromatin immunoprecipitation with fixed animal tissues and preparation for high-throughput sequencing**. *Cold Spring Harb Protoc* 2015, **2015**(2):191-199.

4. Langmead B, Salzberg SL: **Fast gapped-read alignment with Bowtie 2**. *Nat Methods* 2012, **9**(4):357-359.

5. Ramirez F, Ryan DP, Gruning B, Bhardwaj V, Kilpert F, Richter AS, Heyne S, Dundar F, Manke T: **deepTools2: a next generation web server for deep-sequencing data analysis**. *Nucleic Acids Res* 2016, **44**(W1):W160-165.

6. Buenrostro JD, Giresi PG, Zaba LC, Chang HY, Greenleaf WJ: **Transposition of native chromatin for fast and sensitive epigenomic profiling of open chromatin, DNA-binding proteins and nucleosome position**. *Nat Methods* 2013, **10**(12):1213-1218.

7. Bolger AM, Lohse M, Usadel B: **Trimmomatic: a flexible trimmer for Illumina sequence data**. *Bioinformatics* 2014, **30**(15):2114-2120.

8. Li H, Handsaker B, Wysoker A, Fennell T, Ruan J, Homer N, Marth G, Abecasis G, Durbin R, Genome Project Data Processing S: **The Sequence Alignment/Map format and SAMtools**. *Bioinformatics* 2009, **25**(16):2078-2079.
